# Distinct herpesvirus resistances and immune responses of three gynogenetic clones of gibel carp revealed by comprehensive transcriptomes

**DOI:** 10.1186/s12864-017-3945-6

**Published:** 2017-07-24

**Authors:** Fan-Xiang Gao, Yang Wang, Qi-Ya Zhang, Cheng-Yan Mou, Zhi Li, Yuan-Sheng Deng, Li Zhou, Jian-Fang Gui

**Affiliations:** 1 State Key Laboratory of Freshwater Ecology and Biotechnology, Institute of Hydrobiology, Chinese Academy of Sciences, Wuhan, 430072 China; 2 University of Chinese Academy of Sciences, Beijing, 100049 China

**Keywords:** Gibel carp, Herpesvirus, Transcriptome, Interferon, Disease resistance, Immune response

## Abstract

**Background:**

Gibel carp is an important aquaculture species in China, and a herpesvirus, called as *Carassius auratus* herpesvirus (*Ca*HV), has hampered the aquaculture development. Diverse gynogenetic clones of gibel carp have been identified or created, and some of them have been used as aquaculture varieties, but their resistances to herpesvirus and the underlying mechanism remain unknown.

**Results:**

To reveal their susceptibility differences, we firstly performed herpesvirus challenge experiments in three gynogenetic clones of gibel carp, including the leading variety clone A^+^, candidate variety clone F and wild clone H. Three clones showed distinct resistances to *Ca*HV. Moreover, 8772, 8679 and 10,982 differentially expressed unigenes (DEUs) were identified from comparative transcriptomes between diseased individuals and control individuals of clone A^+^, F and H, respectively. Comprehensive analysis of the shared DEUs in all three clones displayed common defense pathways to the herpesvirus infection, activating IFN system and suppressing complements. KEGG pathway analysis of specifically changed DEUs in respective clones revealed distinct immune responses to the herpesvirus infection. The DEU numbers identified from clone H in KEGG immune-related pathways, such as “chemokine signaling pathway”, “Toll-like receptor signaling pathway” and others, were remarkably much more than those from clone A^+^ and F. Several IFN-related genes, including *Mx1*, *viperin*, *PKR* and others, showed higher increases in the resistant clone H than that in the others. *IFNphi3*, *IFI44-like* and *Gig2* displayed the highest expression in clone F and *IRF1* uniquely increased in susceptible clone A^+^. In contrast to strong immune defense in resistant clone H, susceptible clone A^+^ showed remarkable up-regulation of genes related to apoptosis or death, indicating that clone A^+^ failed to resist virus offensive and evidently induced apoptosis or death.

**Conclusions:**

Our study is the first attempt to screen distinct resistances and immune responses of three gynogenetic gibel carp clones to herpesvirus infection by comprehensive transcriptomes. These differential DEUs, immune-related pathways and IFN system genes identified from susceptible and resistant clones will be beneficial to marker-assisted selection (MAS) breeding or molecular module-based resistance breeding in gibel carp.

**Electronic supplementary material:**

The online version of this article (doi:10.1186/s12864-017-3945-6) contains supplementary material, which is available to authorized users.

## Background

Gibel carp, also known as silver crucian carp or Prussian carp, has been recognized as a subspecies *Carassius auratus gibelio* of crucian carp (*C. auratus*) [1, 2], and currently as a separate species *C. gibelio* owing to its polyploidization and special multiple reproductive modes [3–8]. Gibel carp was found to be able to reproduce by unisexual gynogenesis [1, 2], bisexual reproduction [4, 9–11], hybrid-similar development mode [8] or even androgenesis [12] in response to sperm from different species or gibel carp clones. Since the application of all-female gibel carp produced by heterologous sperm gynogenesis (termed allogynogenesis) to activate embryo development in the early 1980s [2], gibel carp has become a very important aquaculture species in China and the annual production capacity of crucian carp has increased to 2,912,258 tons in 2015 [13, 14]. In aquaculture, gibel carp seeds are generally produced by allogynogenesis that maintains variety purity and high seed survival rate [14]. The homozygous genetic background of each variety has been confirmed by microsatellites, AFLP profiles and transferrin alleles [12]. Recently, the culture industry has suffered enormous economic losses in main culture areas of Jiangsu province due to the epizootic disease caused by crucian carp herpesvirus (*Ca*HV) [15]. *Ca*HV shows high homology to a variant cyprinid herpesvirus 2 (CyHV-2) strain SY-C1 [16] and induces severe mortality. CyHV-2, also named herpesviral haematopoietic necrosis (HVHN), was initially isolated from goldfish (*C. auratus*) in Japan [17].

Diverse local populations or various gynogenetic clones of gibel carp have been identified in different natural regions of Eurasian continent by karyotypes [18–23], serum transferrin phenotypes [24, 25], RAPD (random amplification polymorphism of DNA) and SCAR markers [9, 20, 26], microsatellite markers [27–32], transferrin allele polymorphism [25, 33–35] or mtDNA control region sequences [25, 31, 32, 35–38]. Significantly, some clone-specific molecular markers have been isolated not only for genetic resource identification, but also for marker-assisted selection breeding. In our lab, a series of gibel carp clones, named A, B, C, D, E et al., were successively discriminated from Shuangfeng reservoir, Dongting lake, Pengze lake and other natural regions. Several improved varieties, such as high dorsal allogynogenetic gibel carp (clone D) and allogynogenetic gibel carp “CAS III” (clone A^+^), have been successfully bred in the past 30 years [4, 10, 14]. Currently, the improved variety clone A^+^ is the most popularly cultured varieties in China and the culture scale has accounted for about 70% of gibel carp culture owing to its excellent growth performance [4, 10, 12, 14]. An artificial clone F with subgenomic incorporation was obtained by cold treatment of the clone E eggs inseminated with bluntnose black bream (*Megalobrama amblycephala* Yin) sperm [19, 39, 40] and had been proliferated by a dozen successive generations of gynogenesis with Xingguo red common carp (*Cyprinus carpio*) sperm stimulation, showing rapid growth and disease resistance. Therefore, it is essential to evaluate the susceptibilities of the leading variety clone A^+^ and candidate variety clone F challenged with *Ca*HV. The resistance or tolerance ability of fish to pathogen is determined by genetic factors [41, 42], and several disease-resistant varieties were successfully bred in rainbow trout (*Oncorhynchus mykiss*) [43, 44] and Japanese flounder (*Paralichthys olivaceus*) [45] through selective breeding. Thus, it is an urgent need of gibel carp culture industry to screen resistant gynogenetic clones as core breeding populations to breed novel variety with enhancing *Ca*HV resistance.

Disease resistance is a complex trait that involves various biochemical processes. Host immune responses play central roles in defensing virus attack and involve in innate and adaptive immune systems, such as pathogen recognition receptors, interferon (IFN)-mediated antiviral response, antigen presentation, inflammatory regulators, immune effectors, and so on [46–48]. A lot of IFN system genes have been identified from cultured *Carassius auratus* blastula (CAB) cells induced by UV-inactivated grass carp hemorrhage virus (GCHV) [47, 49–63], and their expression regulation and antiviral mechanisms have been revealed in vitro [50, 52, 54, 56, 64]. Owing to the complexity of fish antiviral immune responses, it is necessary to gain insights into the nature of antiviral host reactions. RNA-Seq has been proved to be an effective technique to find massive immune-related genes and to better understand the complex interactions between virus and host [65–69]. In this study, we firstly evaluated the susceptibilities of clone A^+^, clone F and a wild clone H against to *Ca*HV challenge. Based on transcriptome analysis, the common defenses and the distinct immune responses among three clones were revealed. Finally, DEUs up-regulated or down-regulated in all three clones or specifically in resistant or susceptible clones were analyzed, and full-scale of expression changes of IFN system genes were investigated. This study provides transcriptomic basis for the mechanism of *Ca*HV resistance and will be beneficial to disease-resistance breeding of gibel carp.

## Results

### Different resistance of three gibel carp clones in response to herpesvirus infection

**Fig. 1:**
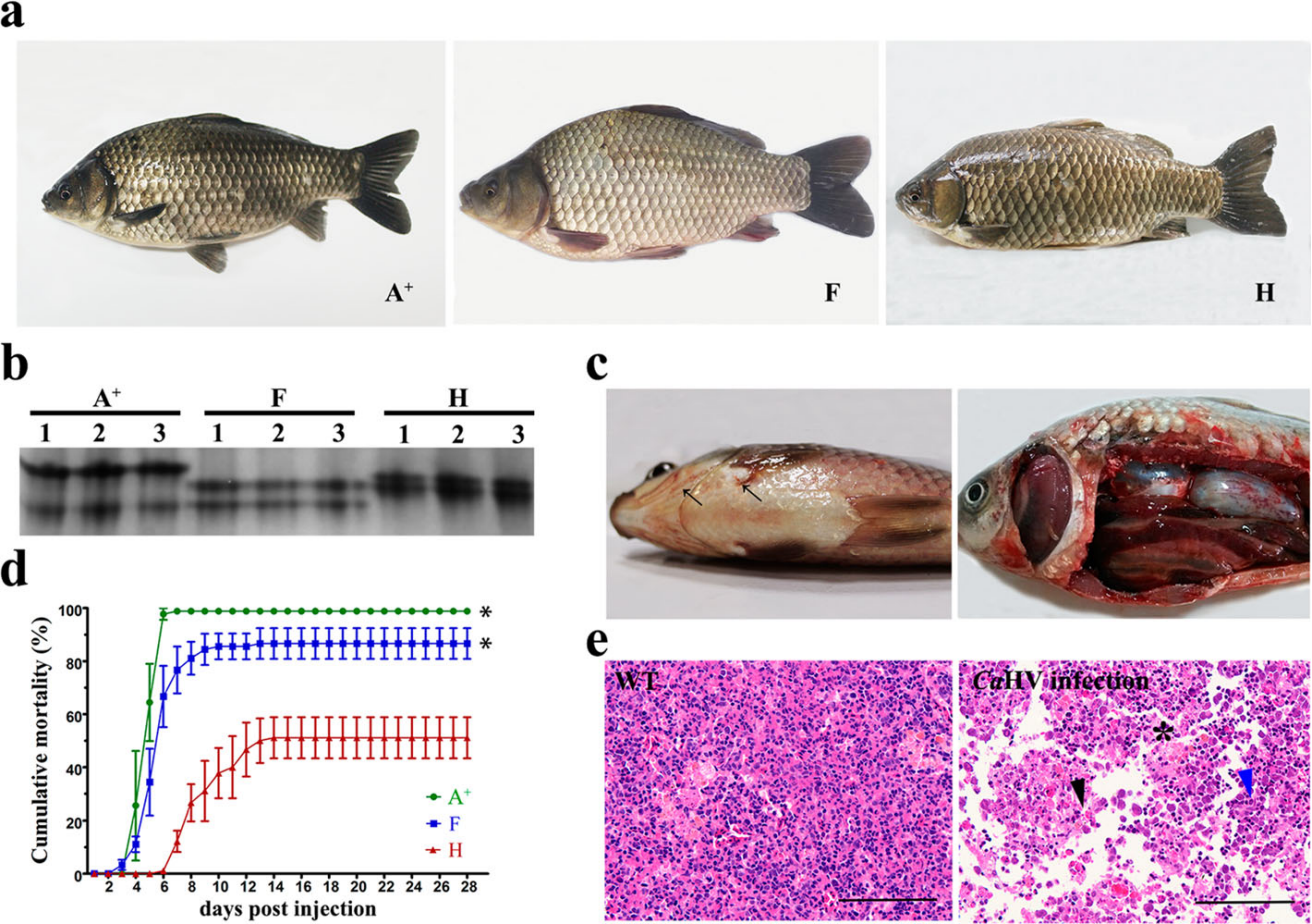
Heterogeneity and different susceptibility to *Ca*HV among three gibel carp clones. **a** Morphological difference among clone A^*+*^, F and H. **b** Transferrin phenotype patterns of three clones with three replications. **c** Symptoms in diseased fish. Hyperemia at the base of the fins and on the abdomen (*black arrows*), bleeding gills and internal organ hemorrhaging. **d** Cumulative mortality after *Ca*HV infection. The values are the mean ± SEM from three replicate tanks. Asterisks (*) indicate significant differences (*P* ≤ 0.05) between clone A^+^ or clone F and clone H. **e** Histopathological photographs of head-kidney from normal and diseased gibel carp (HE). Healthy fish showed no pathological changes, while *Ca*HV infected fish showed that the head-kidney appeared necrotic lesions (*asterisk*), serious vacuolization (*black triangle*), and hypertrophied nuclei with karyorrhexis (*blue triangle*). Scale bars = 100 um

Three gynogenetic clones of gibel carp, clone A^+^, clone F and wild clone H were selected to perform herpesvirus challenge experiments by isolated *Ca*HV. Clones A^+^ and H have spindle-shaped body type, while clone F has a bulge on the anterior back just after the head. Clone A^+^ is silver-black in body color, while clone F is silver-white and clone H is yellow-black (Fig. 1a). Moreover, three clones can be discriminated by different transferrin phenotype pattern [25, 34, 35], and intraclonal homogeneity and interclonal heterogeneity were obvious in these clones (Fig. 1b).

After *Ca*HV infection, the diseased fishes first showed sub-clinical symptoms, including lethargy, anorexia and body color deepened. As the disease progresses, they exhibited bleeding at the base of fins and on abdomen, observed pale gills, internal organ hemorrhaging, pink ascites in abdominal cavity and swollen spleen and kidneys (Fig. 1c). Cumulative mortalities resulted from three independent repetitive experiments were statistically analyzed in these clones. As shown in Fig. 1d, clone A^+^ is most susceptible and clone H is most resistant (*p* < 0.01). The first death of clone A^+^ occurred at 4 days post injection (dpi) and the overwhelming majority (98.89%) died at 7 dpi. In contrast, the death of clone H started at 6 dpi and about half of individuals (51.11%) died at 14 dpi. Clone F was moderately resistant and 86.67% infected individuals died at 13 dpi. Viral loads were evaluated by real-time PCR. The injected fish from clone H had an average viral load of 10^1.88^ and 10^2.16^ particles/ng DNA at 3 dpi and 5 dpi respectively, markedly less than those in clone A^+^ and F which had an average viral load of 10^3.29^ and 10^2.72^ at 3 dpi, and 10^4.49^ and 10^3.04^ at 5 dpi respectively. Histopathological examination was also performed. In comparison with normal tissue of WT, *Ca*HV infection resulted in severe necrotic lesion, serious vacuolization and hypertrophied nuclei with karyorrhexis in head-kidney (Fig. 1e). These results indicate that clone H possesses stronger resistance to herpesvirus infection.

### De novo assembly and functional annotation of head-kidney transcriptomes

**Fig. 2:**
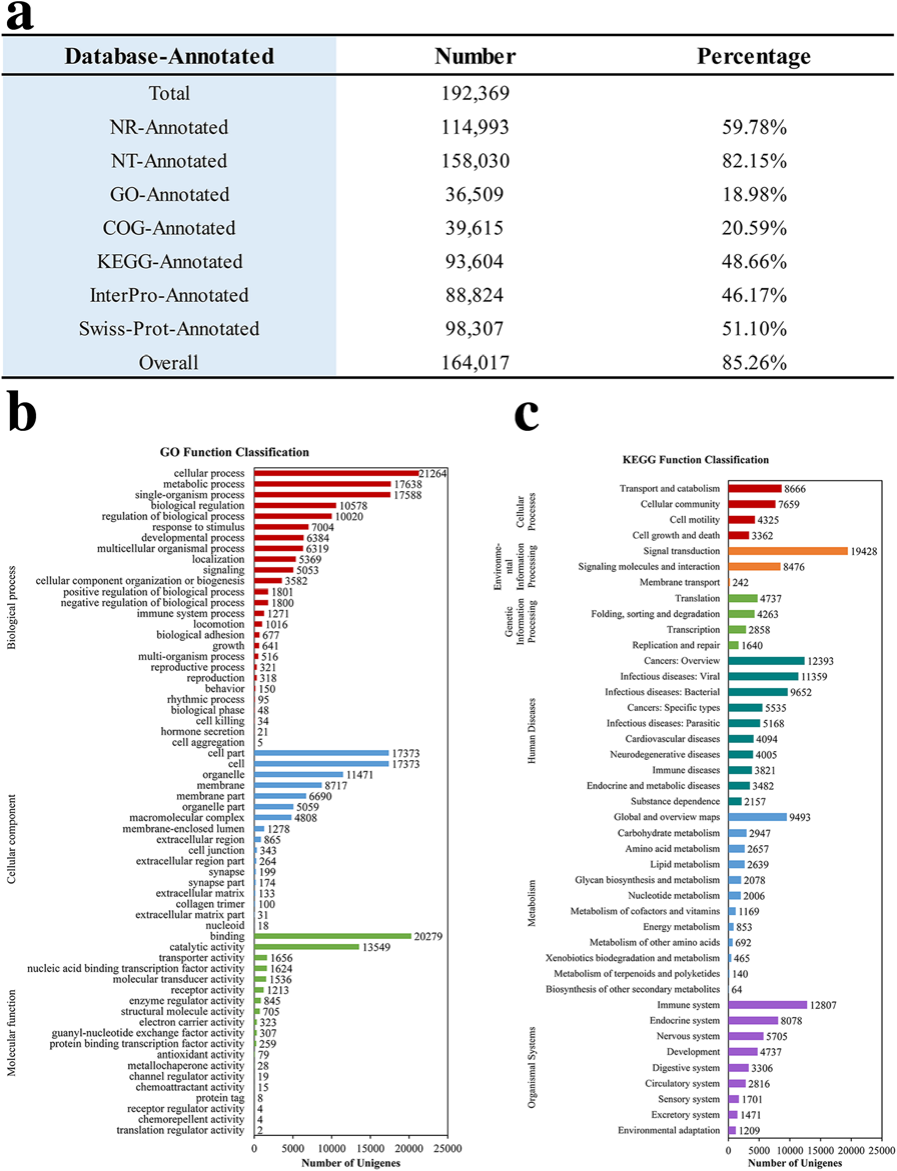
Annotation summary and unigene classification of the gibel carp transcriptome. **a** Overview of unigene annotation. **b** Histogram of GO classification. 36,509 unigenes were assigned to 62 terms grouped into three main categories: biological process (*red bars*), cellular component (*blue bars*) and molecular function (*green bars*). **c** Histogram of KEGG classification. 93,604 unigenes were mapped to 42 terms grouped into six main categories: Cellular Processes (*red bars*), Environmental Information Processing (*orange bars*), Genetic Information Processing (*green bars*), Human Diseases (*dark green bars*), Metabolism (*blue bars*) and Organismal Systems (*purple bars*)

Head-kidney is the main targeting organ of herpesvirus infection and replication [70]. In order to obtain global gene expression profile responding to *Ca*HV infection, we performed comparative head-kidney transcriptome analysis of control (c) and early diseased (d) individuals with sub-clinical symptoms (an average viral load of 10^6^ particles/ng DNA) from clone A^+^, F and H, respectively (Additional file 1: Figure S1). Three biological replicates were carried out. By using Illumina HiSeq™ 4000 platform, a total of 121.45 Gb data were generated from all transcriptome libraries constructed in this study. After removing the reads with low quality or adaptors, 809,509,754 clean reads were obtained and assembled into a total of 192,369 unigenes with an average size of 1342 bp and a N50 value of 2639 bp (Fig. 2a; Additional file 2: Table S1, Additional file 3: Table S2) by Trinity [71] and Tgicl [72]. Subsequently, these unigenes were blasted in seven public databases, including National Center for Biotechnology Information (NCBI) non-redundant protein (NR), NCBI non-redundant nucleotide (NT), Gene Ontology (GO), Clusters of Orthologous Groups of proteins (COG), Kyoto Encyclopedia of Genes and Genomes (KEGG), InterPro and Swiss-Prot database, and overall 164,017 (85.26%) unigenes were identified (Fig. 2a). Of these, 36,509 unigenes were classified into 62 GO terms (Fig. 2a, b). “Cellular process”, “cell part” or “cell” and “binding” were dominant in the category “biological process”, “cellular component” and “molecular function”, respectively. 1271 unigenes were mapped to the term “immune system process”. To identify the pathways involved in physiological function of head-kidney, 93,604 unigenes were mapped to 307 KEGG pathways (Fig. 2a, c). The pathway with most annotated unigenes was “signal transduction” (19,428 unigenes), followed by “immune system” (12,807 unigenes), “cancers: overview” (12,393 unigenes), “infectious diseases: viral” (11,359 unigenes), “infectious diseases: Bacterial” (9652 unigenes), and so on. Therefore, head-kidney triggers violent immune responses when gibel carp is challenged with the herpesvirus *Ca*HV.

### Distinct immune responses to the herpesvirus *Ca*HV infection between susceptible and resistant clones

To elucidate the common defense responses and the molecular mechanisms underlying distinct herpesvirus resistance between susceptible and resistant clones, the head-kidney transcriptome profiles of diseased clone A^+^ (d-A^+^) versus control clone A^+^ (c-A^+^), diseased clone F (d-F) versus control clone F (c-F) and diseased clone H (d-H) versus control clone H (c-H) were compared. A total number of 8772, 8679 and 10,982 DEUs (probability ≥0.8 and relative change ≥2) were identified between the diseased individuals and control individuals from clone A^+^, F and H, respectively (Additional file 4: Figure S2; Additional file 5: Table S3).

GO analysis revealed that the DEUs identified from d-A^+^ vs c-A^+^, d-F vs c-F and d-H vs c-H were categorized into 54, 53 and 54 GO terms respectively, and similar distribution patterns with subtle differences of DEUs were obtained (Additional file 6: Figure S3). Owing to the strikingly more DEUs (>2000) down-regulated in d-H vs c-H (Additional file 4: Figure S2), the numbers of d-H vs c-H DEUs in main GO terms, such as “cellular process”, “single-organism process”, “metabolic process”, “cell”, “cell part”, “binding” and “catalytic activity”, were greater than those identified in d-A^+^ vs c-A^+^ and d-F vs c-F. Of these, 175, 148 and 202 DEUs identified from d-A^+^ vs c-A^+^, d-F vs c-F and d-H vs c-H were mapped to “immune system process”, respectively. Additionally, a few of DEUs were categorized into several GO terms, which were presented only in one clone or two clones, such as “metallochaperone activity” and “hormone secretion” only in d-H vs c-H, “cell killing” in d-F vs c-F and d-H vs c-H, “biological phase” and “rhythmic process” in d-A^+^ vs c-A^+^ and d-H vs c-H, and so on.

**Fig. 3:**
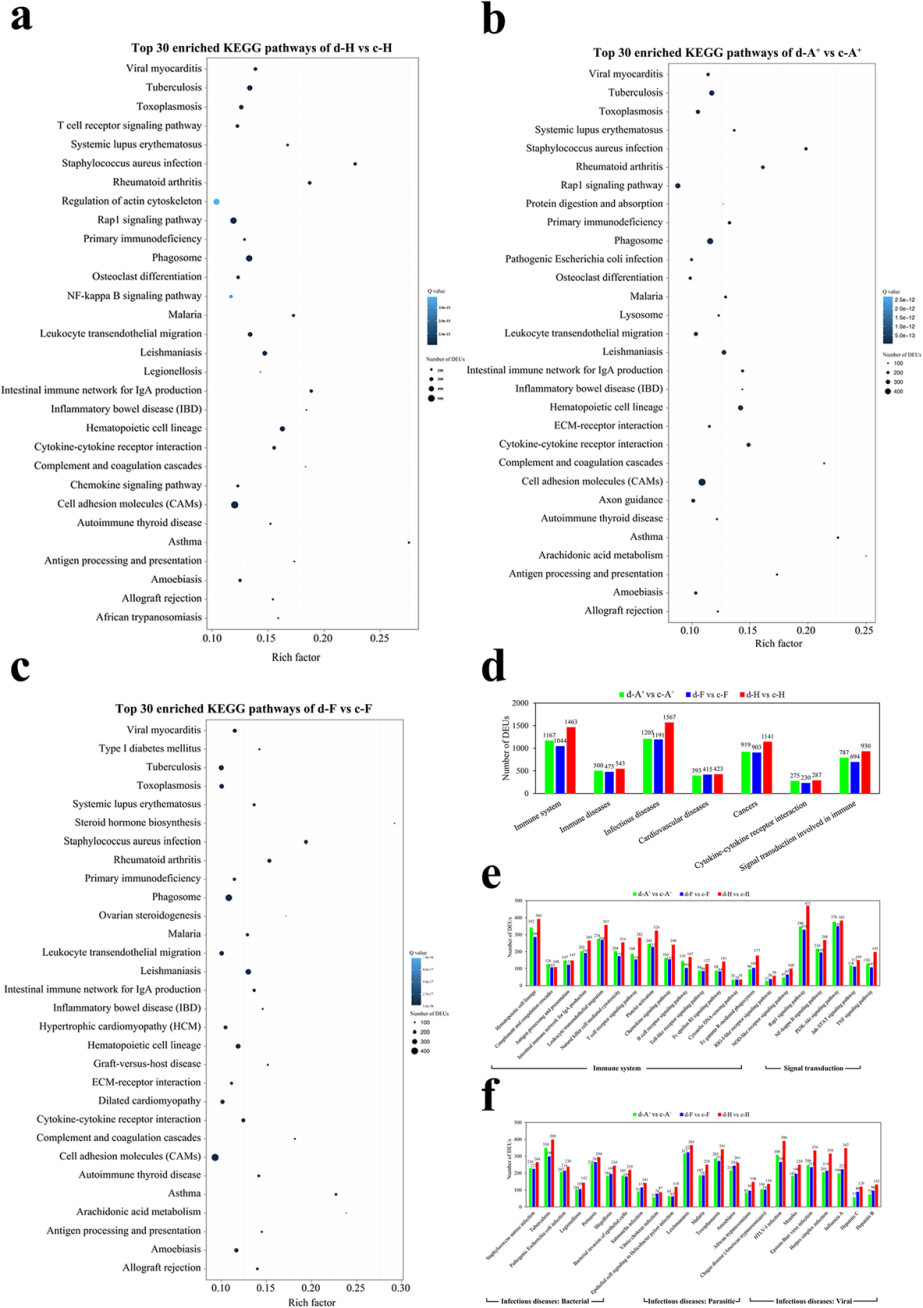
KEGG pathway enrichment analysis in each clone. Top 30 enriched KEGG pathways of DEUs from d-H vs c-H **a**, d-A^+^ vs c-A^+^ **b** and d-F vs c-F **c**. The x-axis indicates the rich factor of each pathway and y-axis shows pathway. The color and size of dot indicates Q value and the numbers of DEUs assigned to the corresponding pathway respectively. **d** The numbers of DEUs in immune-related or disease-related KEGG pathways at level 2. **e** The numbers of DEUs in immune-related KEGG pathways at level 3. **f** The numbers of DEUs in infectious diseases-related KEGG pathways at level 3. d-A^+^ vs c-A^+^, d-F vs c-F and d-H vs c-H are abbreviations of clone A^+^ diseased fishes (d-A^+^) versus clone A^+^ control fishes (c-A^+^), and so on

KEGG pathway mapping revealed that these DEUs were involved in about 300 similar pathways in three clones. Among the top 30 pathways, 26 pathways enriched in d-H vs c-H were associated with “immune system”, “immune diseases”, “infectious diseases”, “cardiovascular diseases” or immune-related pathways in “signaling molecules and interaction” and “signal transduction”, such as “cytokine-cytokine receptor interaction”, “Rap1 signaling pathway” and “NF-kappa B signaling pathway” (Fig. 3a; Additional file 7: Table S4). However, 8 pathways among the top 30 pathways enriched in d-A^+^ vs c-A^+^, including “cell adhesion molecules (CAMs)”, “arachidonic acid metabolism”, “ECM-receptor interaction”, “axon guidance”, “osteoclast differentiation” and so on, did not related to immune responses (Fig. 3b; Additional file 7: Table S4). The differences of enriched pathways between d-H vs c-H and d-A^+^ vs c-A^+^ suggest that differential immune responses occur in susceptible clone and resistant clone.

To reveal the detailed differences of defense responses between susceptible and resistant clones, the numbers of DEUs in KEGG immune-related or disease-related pathways, including “immune system”, “immune diseases”, “infectious diseases”, “cardiovascular diseases”, “cancer”, “cytokine-cytokine receptor interaction” and “signal transduction involved in immune” were calculated. As shown in Fig. 3d, the numbers of DEUs in “immune diseases”, “cardiovascular diseases” and “cytokine-cytokine receptor interaction” were approximately equal among d-A^+^ vs c-A^+^, d-F vs c-F and d-H vs c-H. However, in term “immune system”, “infectious diseases”, “cancer” and “signal transduction involved in immune”, obviously more unigenes in clone H changed their expression levels than in clone A^+^ and F. Subsequently, the term “immune system” were further sub-classified into 16 pathways, such as “hematopoietic cell lineage”, “complement and coagulation cascades”, “antigen processing and presentation”, “Toll-like receptor signaling pathway” and so on, and “signal transduction involved in immune” included “Jak-STAT signaling pathway”, “Rap1 signaling pathway”, “NF-kappa B signaling pathway”, “PI3K-Akt signaling pathway” and “TNF signaling pathway” (Fig. 3e). The numbers of DEUs were approximately equal between two susceptible clones A^+^ and F in all 21 pathways. In contrast, significantly more DEUs were identified from d-H vs c-H than those in d-A^+^ vs c-A^+^ and d-F vs c-F, except “complement and coagulation cascades”, “antigen processing and presentation”, “cytosolic DNA-sensing pathway” and “PI3K-Akt signaling pathway”. The term “infectious diseases” was also subdivided into 10 pathways in “infectious diseases: bacterial”, 6 pathways in “infectious diseases: parasitic” and 7 pathways in “infectious diseases: viral”. Compared to the faint differences of DEUs numbers among three clones in “infectious diseases: bacterial” and “infectious diseases: parasitic”, the number of DEUs in “infectious diseases: viral” identified from d-H vs c-H were remarkably greater than those in d-A^+^ vs c-A^+^ and d-F vs c-F (Fig. 3f), consistent with the herpesvirus nature of *Ca*HV. All the results suggest that distinct immune responses occur between resistant and susceptible gibel carp clones after the herpesvirus *Ca*HV infection.

### Common defense responses of three different clones in response to the herpesvirus *Ca*HV infection

**Fig. 4:**
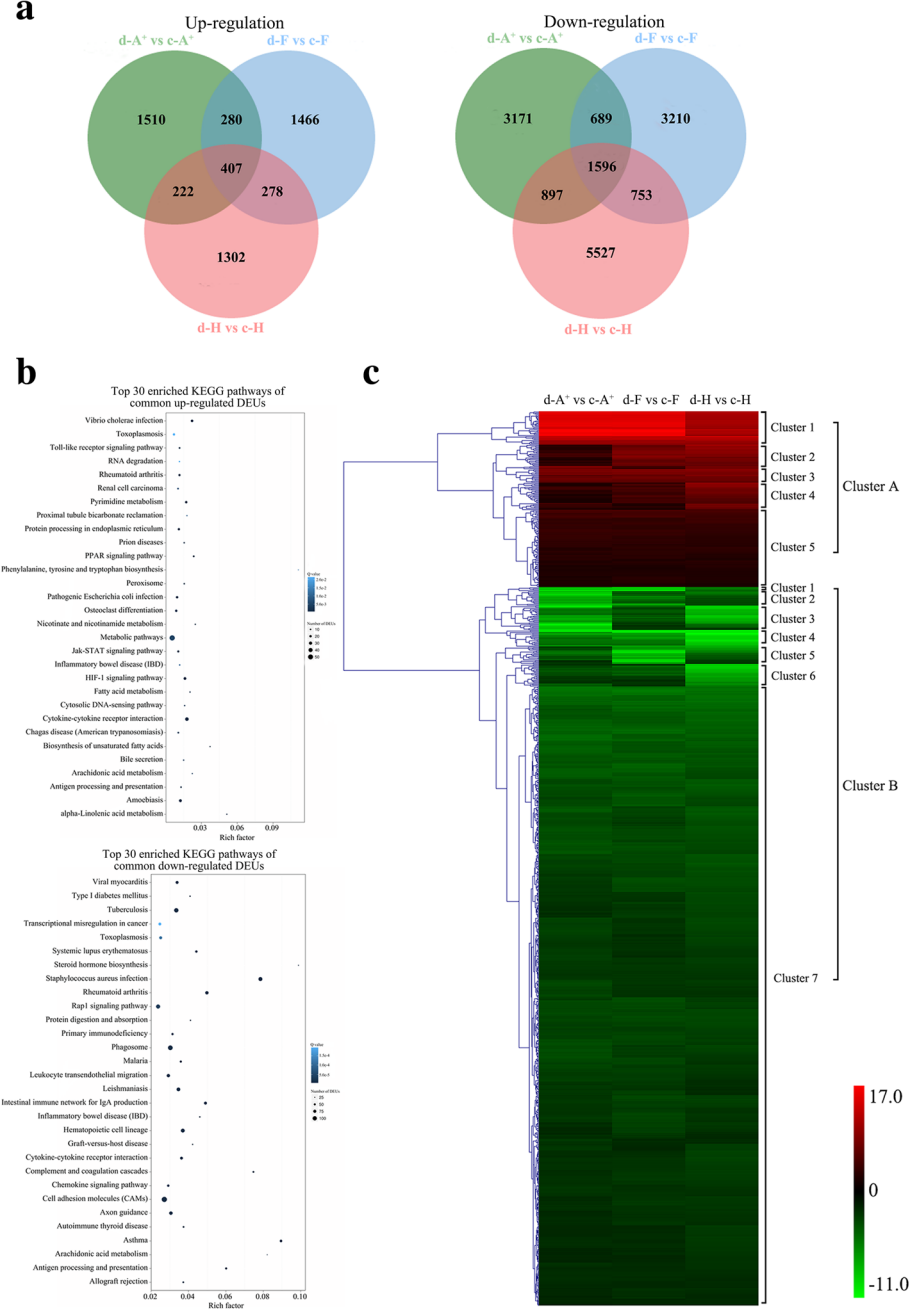
Common DEUs among gibel carp clones. **a** Venn diagram of up- or down-regulated DEUs among three comparisons: d-A^+^ vs c-A^+^ (*green circle*), d-F vs c-F (*blue circle*) and d-H vs c-H (*red circle*). **b** Top 30 enriched KEGG pathways of commonly up- or down-regulated DEUs in clone A^+^, F and H. The x-axis indicates the rich factor of each pathway showed at y-axis. The color and size of dot indicates Q value and the number of DEUs assigned to the corresponding pathway respectively. **c** Heatmap of commonly changed DEUs in three clones with hierarchical clustering calculated by log_2_ fold change values and the main clusters were lined out on the right. Up-regulated DEUs are shown in red and down-regulated DEUs in green

The shared DEUs among d-A^+^ vs c-A^+^, d-F vs c-F and d-H vs c-H were integrated. 5465 up-regulated and 15,843 down-regulated DEUs were obtained. Among these DEUs, 407 and 1596 DEUs were up-regulated or down-regulated in all three gibel carp clones after *Ca*HV infection (Fig. 4a). The pathway with most annotated unigenes of commonly up-regulated DEUs in KEGG was “cytokine-cytokine receptor interaction” which indicates cytokines play a vital role in resistance to *Ca*HV invasion. In addition, “Toll-like receptor signaling pathway”, “cytosolic DNA-sensing pathway” and “Jak-STAT signaling pathway”, were also observed in the top 30 enriched KEGG pathways. In contrast, “complement and coagulation cascades”, “intestinal immune network for IgA production”, “hematopoietic cell lineage”, “leukocyte transendothelial migration” and “chemokine signaling pathway” were found in the top 30 enriched KEGG pathways of commonly down-regulated DEUs (Fig. 4b; Additional file 8: Table S5). The results suggest that *Ca*HV infection result in the activation of IFN system genes and the down-regulation of immune related genes involved in complement system.

Approximately 38.3% (156/407) up-regulated DEUs or 34.5% (551/1596) down-regulated DEUs in all three clones were mapped into KEGG immune-related pathways or disease-related pathways (Additional file 8: Table S5). A lot of IFN system genes, such as *IFN* (*IFNγ*, and *IFNphi2*), interferon-induced protein (*IFI35*, *IFI56* and *IFI58*), IFN antiviral effectors (*Mx1*, *Mx3* and *MxE*), IFN-stimulated genes (ISGs) (*viperin* and *Gig1*), interferon-induced guanylate-binding protein *GBP1*, and immunity-related GTPase family *IRGE4*, interleukins (*IL-6a* and *IL-11*), chemokines (*CXCL-C1c*, *CCL-C5a* and *CXCl1*) and tumor necrosis factor superfamily members (*tnfrsf1a*, *tnfrsf5*, *tnfrsf11b*, *tnfrsf12a*, *tnfrsf13b* and *tnfrsf21*) were up-regulated in all three clones. Among the 551 down-regulated DEUs, a lot of unigenes belonged to “complement and coagulation cascades”, such as complement factor properdin (*CFP*), *CFB*, *CFD*, *CFI*, *CFHR3*, *CFHR-1*, *C3*, *C4–1*, *C5–2*, *C6*, *C7*, complement C3b/C4b receptor 1 (*CR1*), or were clustered into “antigen processing and presentation”, such as different alleles of major histocompatibility complex (MHC) class II alpha or beta antigen, MHC class II transactivator (*CIITA*) isoform X2 and isoform X3. Additionally, the expression of several cluster of differentiation (CD) antigens (*CD3*, *CD4*, *CD9*, *CD44*, *CD59*, *CD79b* and *CD209*), C-C chemokine receptors (*CCR4*, *CCR5* and *CCR7*), interleukin receptors (*IL1R*, *IL2Rβ*, *IL6Rα*, *IL13Rα2*), IFN system genes, including *IFNphi4*, *IFI30*, dsRNA signal sensing factor (*TLR3* and *TLR25*) and interferon regulator factor (*IRF2*, *IRF4*, *IRF5* and *IRF8*), were decreased after *Ca*HV infection.

Hierarchical clustering of the common DEUs was classified into two distinct clusters (A and B) according to the different changes among three clones (Fig. 4c). Cluster A included the commonly up-regulated DEUs and was sub-divided into five distinct clusters. The DEUs in cluster 1 were highly up-regulated in d-A^+^ vs c-A^+^ and d-F vs c-F, in which the vast majority of the DEUs were annotated as intestinal mucin-2. The DEUs in cluster 2 and 4 showed relatively more increases in resistant clone H than in susceptible clone A^+^, including *IFNphi2*, *IFI56*, *IFI58*, *Mx1*, *Mx3*, *viperin*, *Gig1*, *IRGE4*, *IL-6a* and E3 ubiquitin-protein ligase *TRIM39*, which might be important candidate resistant genes to *Ca*HV for further studies. The DEUs in cluster 3 displayed relatively higher increase levels in d-F vs c-F than in d-A^+^ vs c-A^+^ and d-H vs c-H, and the DEUs in cluster 5 showed similar increase levels among three clones after *Ca*HV infection. The commonly down-regulated DEUs were categorized into seven distinct clusters in cluster B, in which cluster 7 included the vast majority of DEUs showing similar a little reduction among three clones after *Ca*HV infection. Cluster 4 contained the DEUs highly down-regulated, such as *IRF5*, *IL6R*, *CFD*, *c-lectin* and so on. The DEUs in cluster 2, 5 and 6 displayed the most expression decreases in clone A^+^, F and H respectively. The representative genes in cluster 6 included *CCR7*, *CD59*, leukotriene B4 receptor *Ltb4r1*, and so on. The DEUs in cluster 1 or 3 exhibited more decrease in clone A^+^ and F than clone H or in clone A^+^ and H than clone F, respectively.

### Specifically changed DEUs and differential immune pathways of three different clones in response to the herpesvirus *Ca*HV infection

**Fig. 5:**
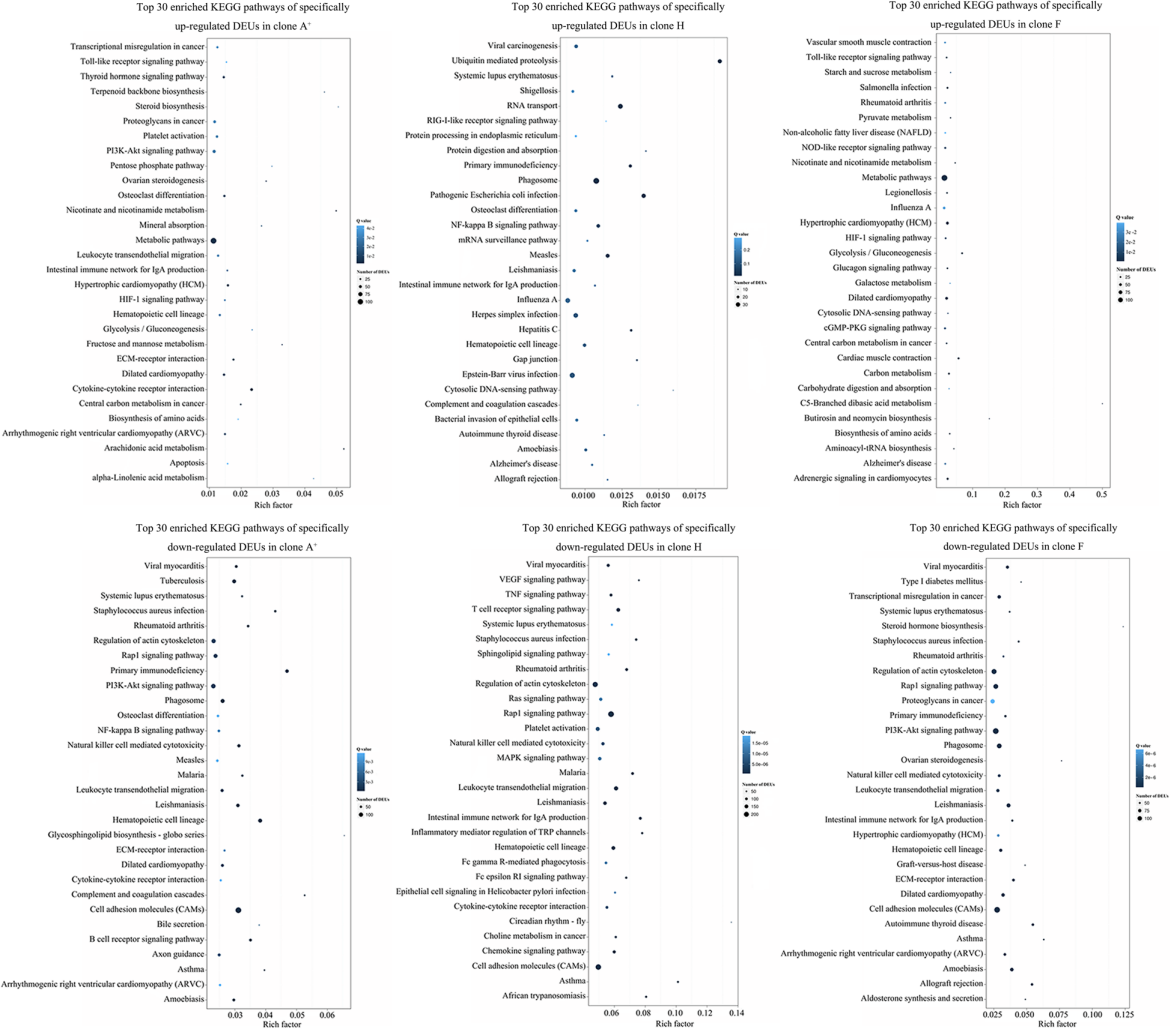
KEGG pathway enrichment analysis of unique DEUs in clone A^+^, H or F, respectively. Top 30 enriched KEGG pathways of specifically up- or down-regulated DEUs in clone A^+^, H or F. The x-axis indicates the rich factor of each pathway. The color and size of dot indicates Q value and the number of DEUs assigned to the corresponding pathway respectively

As shown in Fig. 4a, 4681 (1510 up-regulated and 3171 down-regulated), 4676 (1466 up-regulated and 3210 down-regulated) and 6829 (1302 up-regulated and 5527 down-regulated) changed their expression levels in clone A^+^, F and H respectively after the herpesvirus *Ca*HV infection (Fig. 4a; Additional file 9: Table S6). The KEGG mapping analysis showed the differential enriched pathways among these DEUs (Fig. 5; Additional file 8: Table S5). The DEUs specifically upregulated in susceptible clone A^+^ were chiefly mapped to “intestinal immune network for IgA production”, “hematopoietic cell lineage”, “platelet activation”, “leukocyte transendothelial migration”, “PI3K-Akt signaling pathway”, “HIF-1 signaling pathway”, “cytokine-cytokine receptor interaction” and “Toll-like receptor signaling pathway”, while the DEUs specifically upregulated in resistant clone H were mainly mapped to “cytosolic DNA-sensing pathway”, “hematopoietic cell lineage”, “intestinal immune network for IgA production”, “complement and coagulation cascades”, “RIG-I-like receptor signaling pathway” and “NF-kappa B signaling pathway”. The DEUs specifically upregulated in clone F were mapped to “Toll-like receptor signaling pathway”, “NOD-like receptor signaling pathway” and “cytosolic DNA-sensing pathway”. Interestingly, there exist remarkable differences in terms of down-regulated DEUs of three gynogenetic clones. Among the top 30 KEGG pathways enriched by down-regulated DEUs specific to resistant clone H, 9 pathways were associated with “immune system”. However, only 4 and 5 KEGG pathways enriched by down-regulated DEUs specific to clone F and A^+^ were clustered into “immune system”. Besides the shared 3 pathways (e.g., “leukocyte transendothelial migration”, “hematopoietic cell lineage” and “natural killer cell mediated cytotoxicity”) in three gynogenetic clones and “intestinal immune network for IgA production” shared in clone F and A^+^, other 2 pathways, “B cell receptor signaling pathway” and “complement and coagulation cascades”, were down-regulated in susceptible clone A^+^, while 5 different pathways (e.g., “T cell receptor signaling pathway”, “chemokine signaling pathway”, “Fc epsilon RI signaling pathway”, “platelet activation” and “Fc gamma R-mediated phagocytosis”) were observed to be enriched in resistant clone H. Additionally, 229 and 102 DEUs specifically down-regulated in clone H mapped to “Rap1 signaling pathway” and “cytokine-cytokine receptor interaction”, respectively. The differences of enriched pathways reveal a marked differences in immune response of gibel carp three gynogenetic clones.

**Fig. 6:**
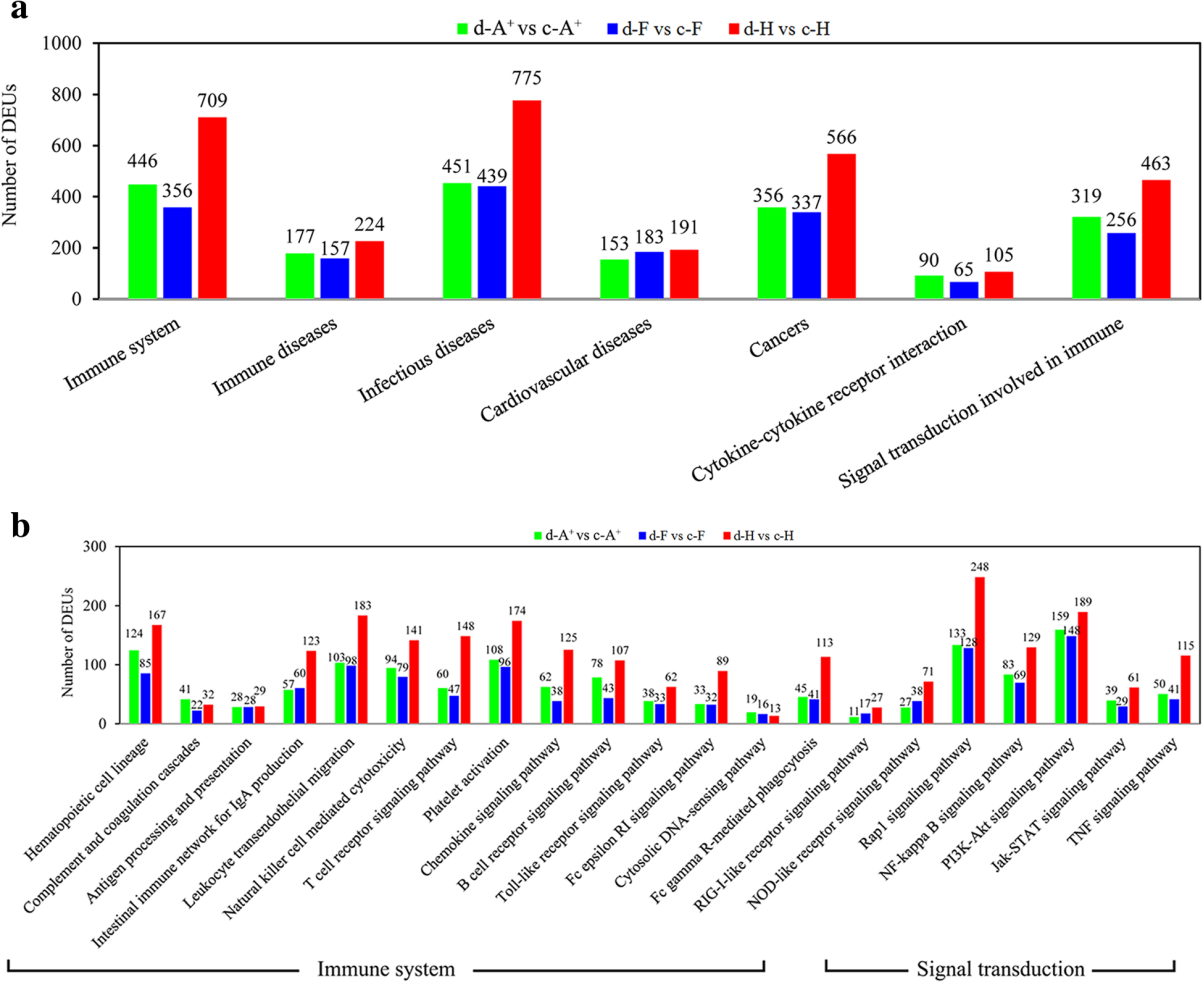
DEUs in corresponding KEGG pathways. **a** Histogram of the numbers of specifically changed DEUs in immune-related or disease-related KEGG pathways at level 2. **b** Histogram of the numbers of specifically changed DEUs in immune-related KEGG pathways at level 3

The numbers of these DEUs in KEGG immune-related or disease-related pathways were also calculated. As shown in Fig. 6a, obviously more DEUs in “immune system”, “infectious diseases”, “cancer” and “signal transduction involved in immune” were detected in clone H than in clone A^+^ and F. Consistent with the results of all DEUs identified from d-A^+^ vs c-A^+^, d-F vs c-F and d-H vs c-H (Fig. 3d), the number of DEUs unique in resistant clone was greater than those in susceptible clone A^+^ and F, except “complement and coagulation cascades”, “antigen processing and presentation”, “cytosolic DNA-sensing pathway” and “PI3K-Akt signaling pathway” (Fig. 6b).

Among these DEUs specific to d-H vs c-H, approximately 15.1% (196/1302) up-regulated DEUs and 21.5% (1191/5527) down-regulated DEUs were mapped into KEGG immune-related pathways or disease-related pathways (Additional file 8: Table S5). A serial of IFN system genes, such as interferon-inducible and dsRNA-dependent protein kinase *PKR*, *PKZ*, *IFI27-like*, *IRF7*, stimulator of interferon *MAVS*, and interferon-induced protein with tetratricopeptide repeat *IFIT*, specifically increased their expression after *Ca*HV infection, while several other IFN system genes or immune-related genes, such as Toll-like receptor 5 (*TLR5*), Janus kinase 2 (*JAK2*), and interferon induced transmembrane protein 1 (*IFITM1*), uniquely decreased their expression. In addition, after exposing to *Ca*HV, immunoglobulin (*Ig*) light chain, *Igh*, *Ighm*, *Ighv*, *Igll1*, polymeric immunoglobulin receptor-like (*pigrl*) and a novel immune type receptor with Ig domains specifically raised their expression, while CD antigens (*CD8*, *CD11*, *CD18*, *CD22*, *CD80*, *CD81*, *CD247* and *CD276*), C-C chemokine receptors (*CCR3.1*, *CCR9* and *CCR10*) and interleukin receptors (*IL4R*, *IL7Rα* and *IRAK4*) uniquely reduced their expression.

Among these DEUs specific to d-F vs c-F, approximately 20.1% (293/1466) up-regulated DEUs and 15.7% (505/3210) down-regulated DEUs were clustered into KEGG immune-related pathways or disease-related pathways (Additional file 8: Table S5). Two IFN system genes (*IFNphi3* and *IFI44-like*) and interleukin *IL8* specifically raised their expression in clone F after *Ca*HV infection, while CD antigen *CD40* and chemokine *CCL-c11a* decreased their expression. Among these DEUs specific to d-A^+^ vs c-A^+^, approximately 18.1% (274/1510) up-regulated DEUs and 19.8% (628/3171) down-regulated DEUs were mapped into KEGG immune-related pathways or disease-related pathways (Additional file 8: Table S5). Different from a lot of IFN system genes specifically up-regulated in resistant clone H, only one IFN system gene, *IRF1* was identified from the DEUs uniquely upregulated in susceptible clone A^+^. A lot of apoptosis or death related genes, such as tumor suppressor protein *p53*, *bcl-2-like*, *Mcl1b*, *natural killer cell receptor 2B4* (*CD244*)*-like isoform X1, X2 and X3*, tumor necrosis factor receptor superfamily member *tnfrsf9*, death-associated protein kinase *dapk3* and hematopoietic death receptor *FAS*, specifically increased their expression after *Ca*HV infection. The results suggest that the susceptible clone A^+^ failed to protect against virus and evidently induced apoptosis or death. Additionally, interleukins (*IL10* and *IL22*), interleukin receptors (*IL6Rβ-like* and *IL21R*), chemokines (*CXC11*, *CCL-C25a* and *CCL-C24j*) and CD antigens (*CD27* and *CD48*) specifically raised their expression. In addition, several IFN system genes (*IRF9* and *IFITM3*), complements (*B/C2-A2*, *B/C2-A3*, *C3-H1*, *C3-S*, *C9* and *CHFL4*), chemokines (*CXCb2*, *CXCL-C1* and *CCL4*), C-C chemokine receptors (*CCRL2*, *CXCR1* and *CXCR3*), CD antigens (*CD2*, *CD8beta-1a* and *CD86*) and interleukin receptors (*Il1r1*, *Il3r2* and *IL2Rγ*) were identified from the down-regulated DEUs specific to d-A^+^ vs c-A^+^.

### Significantly changed IFN system genes of three different clones in response to the herpesvirus *Ca*HV infection

**Fig. 7:**
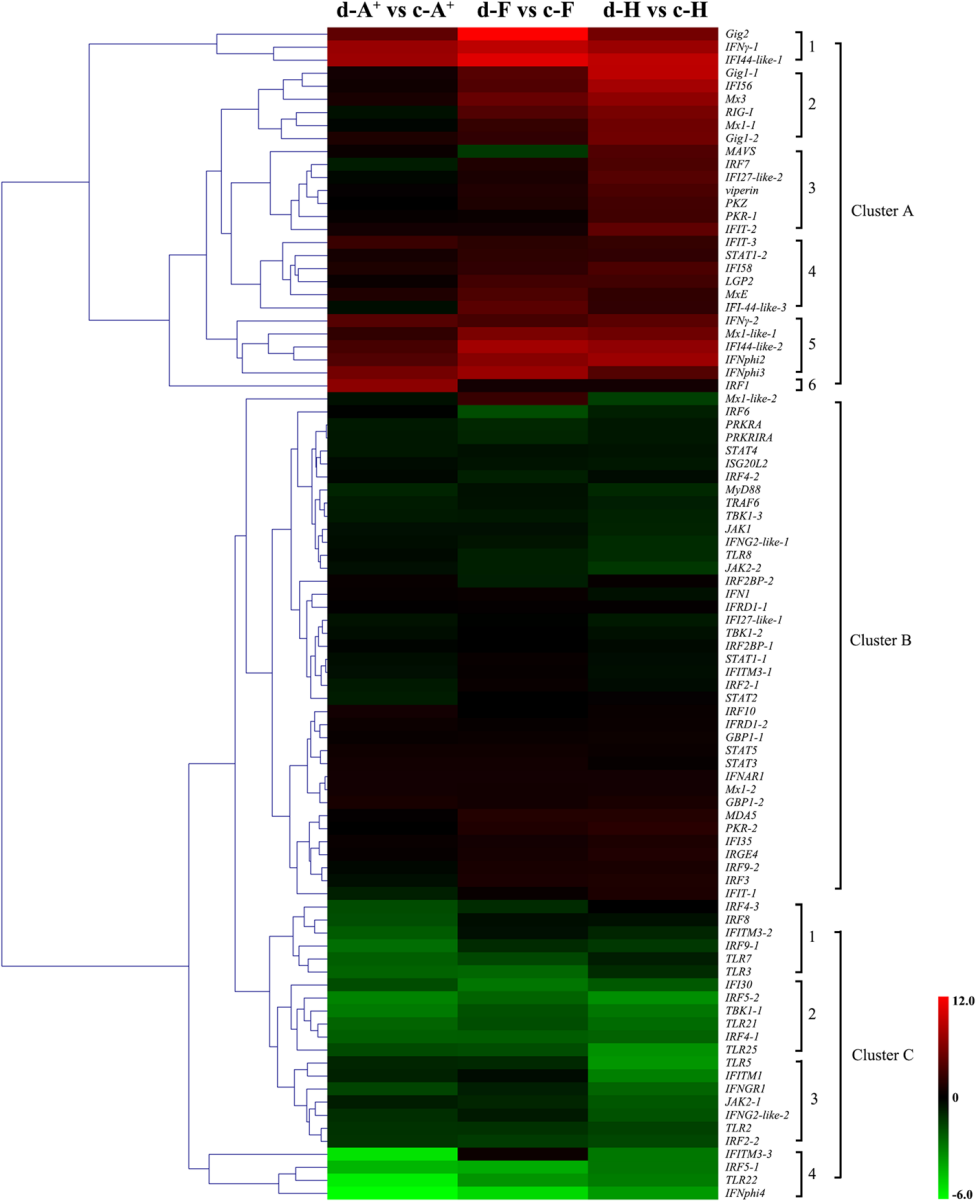
Heatmap of IFN system genes. Hierarchical clustering is calculated by log_2_ fold change values and the main clusters are lined out on the right. Up-regulated DEUs are shown in red and down-regulated DEUs in green

To globally investigate the expression changes of IFN system genes, we searched them from the de novo transcriptome assembly data (Additional file 3: Table S2). The DEUs annotated as IFN system genes and exhibiting representative expression pattern were selected to perform hierarchical clustering analysis. As shown in Fig. 7, IFN system genes were categorized into three distinct clusters (A, B and C) according to their expression change status. The genes in cluster A and cluster C displayed contrasting profiles of gene expression after *Ca*HV infection. The cluster A was sub-divided into 6 clusters. The DEUs in cluster 1, 4 and 5, including *IFNγ-1*, *IFNγ-2*, *IFNphi2*, *IFNphi3*, *Mx1-like-1*, *IFI44-like-1*, *IFI44-like-2*, *IFI44-like-3* and *Gig2*, remarkably or relatively increased their expression levels among three clones. Interestingly, *IFNphi3*, *IFI44-like-1*, *IFI44-like-2*, *IFI44-like-3* and *Gig2* showed the highest increase in clone F after *Ca*HV infection. Cluster 2 and 3 consisted of the resistant-related genes, such as *viperin*, *PKR-1*, *PKZ*, *IRF7*, *Mx1–1*, *IFI56*, *Mx-3*, *Gig1–1*, *Gig1–2*, *RIG-I*, and so on, which displayed relatively more increases in resistant clone H than in susceptible clone A^+^ or specifically increased only in resistant clone H. *IRF1* was very special and uniquely raised its expression only in susceptible clone A^+^. The cluster C was sub-divided into 4 clusters. The DEUs in cluster 2 and 4 reduced their expression among three clones, consisting of *IFNphi4*, *IFI30*, *TBK1–1*, *IRF5–1*, *IRF5–2* and so on. Cluster 2 included the DEUs displaying relatively more decreases in resistant clone H than in susceptible clone A^+^, while the genes in cluster 3 showed remarkably more reductions in resistant clone H, such as *JAk2–1*, *TLR2*, *TLR5*, *IFITM1*, and so on. The genes in cluster B, including *IFN1*, *IRF2*, *IRF3*, *IRF6*, *STAT1, STAT2*, interferon alpha/beta receptor *IFNAR1*, interferon-inducible double stranded RNA dependent inhibitor *PRKRIRA*, interferon-inducible double stranded RNA dependent activator *PRKRA*, interferon stimulated exonuclease gene *ISG20L2*, interferon-related developmental regulator *IFRD1*, TANK-binding kinase *TBK1*, melanoma differentiation-associated gene *MDA5*, myeloid differentiation primary-response protein *MyD88*, and so on, showed little change after *Ca*HV infection among three clones.

## Discussion

Currently, the production capacity of gibel carp contributes to approximate 10% of Chinese freshwater aquaculture production [14]. Owing to the highly intensifying and seriously crowding of monoculture, gibel carp has been suffered by a series of pathogens [73]. Since 2009, an epizootic with acute gill hemorrhages and high mortality has outbroken in cultured gibel carp [74]. The complete genome sequencing of the virus isolated from the tissues of diseased gibel carp showed the *Ca*HV was most closely related to CyHV-2 [15]. CyHV-2 was first reported in 1992 as a pathogen of goldfish in Japan [17, 75], then was identified from goldfish in many countries, such as USA [76, 77], Australia [78], UK [79] and New Zealand [80]. By using PCR assay, CyHV-2 was first detected in gibel carp in Hungary [81]. Recently, it has been detected in many main culture regions of gibel carp [74] and caused huge economic loss. The experimental infection of indigenous Cyprininae species in Japan, such as ginbuna *C. auratus langsdorfii*, nagabuna *C. auratus buergeri*, nigorobuna *C. auratus grandoculis* and common carp, suggested that Japanese *Carassius* fish species possess different ability to depress the replication of CyHV-2 [82]. To obtain gynogenetic clones with strong resistance to *Ca*HV, we evaluated the susceptibilities of a leading variety A^+^ in China, a candidate variety F and wild clones in responsive to *Ca*HV. The cumulative mortalities (Fig. 1d) and viral loads in infected fishes both indicate wild clone H has higher resistance to *Ca*HV than clone A^+^ and F and can be used as core breeding populations to breed novel variety with enhancing *Ca*HV resistance.

In the battle between host and virus, the ability of host to mount a strong immune response is crucial for host survival [83, 84]. In fish, innate protective mechanisms include IFN, complement and cell-mediated cytotoxic activities [85]. As the first defense line of host, IFN system plays a vital role in resistance to viral infection [47]. From UV-inactivated GCHV-infected CAB cells, about 20 IFN system genes, such as *TLR3*, *IFN*, *IRF1* [49], *IRF3* [50], *IRF7* [51], *IRF9* [52], signal transducer and activator of transcription 1 (*STAT1*) [53], mediator of IRF3 activation (*MITA*) [54], *Mx1* and *Mx2* [55], *PKR*, *PKZ* [56], *PKR-like* [57], *IFI58* and *IFI56* [58], *Gig1* and *Gig2* [59], *ISG15–1* and *ISG15–2* [60], *viperin* [61], ubiquitin-specific protease 18 (*USP18*) [62] were identified. Similar to mammalian [86], fish initiates IFN response through recognizing viral products via TLRs and retinoic acid-inducible gene I (RIG-I)-like receptors (RLRs), then triggers IRF3/7-dependent IFN response [10, 47, 87–89]. Interestingly, unique mechanisms underlying IRF3/7-dependent fish IFN response and auto-regulation of fish IFN gene expression were observed in UV-inactivated GCHV-infected CAB cells [47, 49, 50]. After exposing to *Ca*HV, several pathways, “cytokine-cytokine receptor interaction” including IFNs, interleukins and chemokines, “Toll-like receptor signaling pathway” and “Jak-STAT signaling pathway” responsible for recognizing viral production or activating ISGs, were up-regulated in all three gibel carp clones (Fig. 4b; Additional file 8: Table S5), which clearly indicates that IFN response involves in common immune reaction of gibel carp to *Ca*HV invasion.

Consistence with KEGG pathway mapping analysis, a lot of IFN system genes, including *IFNγ*, *IFNphi2*, *IFI35*, *IFI44-like*, *IFI56*, *IFI58*, *Mx1*, *Mx3*, *MxE*, *viperin*, *Gig1*, *Gig2* and *GBP1*, were up-regulated in all three clones (Additional file 9: Table S6), indicating that IFN-mediated innate immune response is one of major common immune defenses of gibel carp to *Ca*HV. Significantly, most of these IFN system genes, such as *viperin*, *PKR-1*, *PKZ*, *IRF7*, *Mx1–1*, *IFI56*, *Mx-3*, *Gig1–1*, *Gig1–2*, *RIG-I*, and so on, showed relatively more increases in resistant clone H than in susceptible clone A^+^ or specifically increased their expression only in resistant clone H (Fig. 7). The similar dynamical expression changes of IFN system genes were also observed between resistant and susceptible Atlantic salmon (*Salmo salar*) families challenged with infectious pancreatic necrosis virus (IPNV) [90, 91]. At 1, 5 and 21 dpi, the expression levels of *IFN*, *Mx1* and *PKR* in head-kidney of resistant family were higher than those in susceptible family [90]. In another resistant and susceptible full-sibling families of Atlantic salmon, *IFNα* and *IFNγ* increased rapidly at 1 dpi, then dropped to the basal values at 5 dpi in susceptible family, while their expression slightly raised and then maintained in resistant family at 5 dpi which were higher than those in susceptible family [91]. Conversely, a completely different immune reaction was observed in other resistant and susceptible families of Atlantic salmon. The vast majority of innate immune response genes, including *IFNα*, *IFNγ*, *Mx*, *ISG15*, *viperin* and *Gig2*, had higher expression in whole fry of susceptible family than in resistant family [92]. These different dynamical changes of IFN system genes among different Atlantic salmon families might be due to different tissues used (head-kidney in the two formers and whole fry in the latter) or different families [92], which indicated that fish immune response against virus is complex and possesses a species-specific or tissue-specific manner. Further studies are necessary to verify the changes of these important DEUs identified in this study at different stages after infection and to reveal their regulative mechanisms behind differential expression between gibel carp resistant and susceptible clones. And the association between single nucleotide polymorphisms (SNPs) of immune-related genes (*IL-10a* and *MHC class IIB*) and resistance to the cyprinid herpesvirus-3 (*Cy*HV-3) had been revealed in common carp [93, 94]. Thus, the differential DEUs, especial IFN system genes showing up-regulation expression in resistant clone H after *Ca*HV infection, might be a key factor for its stronger resistance to *Ca*HV and their allelic variation related to herpesvirus resistance will be able to use as molecular module markers for disease-resistance breeding in gibel carp.

Inflammatory response is crucial for protection against pathogens, and causes significant tissue damage, which involved in cytokines. Besides IFN, cytokines also include chemokines, interleukin, tumor necrosis factor super-families (TNF), colony stimulating factors and so on [95, 96]. IL-6 superfamily is produced in the early stages of infection and involves in diverse immune and neuroendocrine processes, including the regulation of lymphocyte and monocyte differentiation, migration of leukocytes towards the sites of inflammation and chemokine secretion [97]. In this study, the pathway with most annotated unigenes of commonly up-regulated DEUs in KEGG was “cytokine-cytokine receptor interaction”. Two members of IL6 superfamily, *IL6* and *IL11*, raised their expression in all three clones (Additional file 9: Table S6). Significantly, *IL6* increased more in resistant clone H than in susceptible clone A^+^. The up-regulation of *IL6* was also observed in Japanese pufferfish (*Fugu rubripes*) [98], rainbow trout [99], Japanese flounder [100], gilthead seabream (*Sparus aurata*) [101], and European sea bass (*Dicentrarchus labrax*) [102]. After LPS, poly I:C or pathogen infection, Japanese flounder, rainbow trout and gibel carp *IL11* also increased expression after challenge with viral [74, 103, 104]. Rainbow trout recombinant IL6 protein can promote macrophage growth in culture and induce up-regulation of antimicrobial peptide [105]. The immunoglobulin production is mainly regulated by IL6 [106]. Fugu (*Takifugu rubripes*) or orange-spotted grouper recombinant IL6 protein can induce the production of IgM [107, 108]. In accord with the higher expression of IL6 in resistant clone than in susceptible clone, a lot of DEUs annotated as immunoglobulin were identified from the file including up-regulated DEUs specific to d-H vs c-H (Additional file 9: Table S6). In contrast, *IL10* and *IL22* were found in the up-regulated DEUs specific to d-A^+^ vs c-A^+^ (Additional file 9: Table S6). *IL10* was initially identified as a cytokine synthesis inhibitory factor [109] and had been reported to down-regulate *IL6* expression [110]. Indian major carp (*Labeo rohita*) recombinant IL10 protein induced down-regulation of most pro-inflammatory cytokines and up-regulation of natural killer enhancing factors [111]. The higher up-regulation of *IL10* in clone A^+^ suggests a weaker immune response. Additionally, *IRF1* specifically raised its expression level only in susceptible clone A^+^ after *Ca*HV infection. IRF-1 promotes apoptosis following DNA damage [112]. Consistent with upregulation of *IL10* and *IRF-1* in susceptible clone A^+^, a lot of apoptosis or death related genes, such as *p53*, *bcl-2-like*, *Mcl1b*, *CD244*, *dapk3* and *FAS*, showed more increases or specifically raised their expression in clone A^+^ after exposing to *Ca*HV (Additional file 9: Table S6).

After exposure to *Ca*HV, a lot of DEUs mapped to “complement and coagulation cascades” were down-regulated in all three gibel carp clones (Fig. 4b; Additional file 8: Table S5). The relationship between virus and complements is very complicated [113–115]. The complement system plays multiple roles in defending virus evasion, including alerting host to the presence of virus, eliminating invading virus, promoting inflammatory response, clearing apoptotic cell and necrotic cell debris, and modulating innate and adaptive immune response [113–120]. In the meantime, virus has evolved multiple strategies to escape the attack of complements, such as expressing mimics of host complement regulators [113–115]. Although the mechanisms of complement activation and evasion have been revealed clearly in mammals [113], the elaborate interactions between complements and viruses in fish are still unknown and the dynamic expression changes of fish components after virus infection are inconsistent. Generally, fish components are up-regulated after virus infection. However, several exceptions indicate complement system might act various immune effecter functions depending on the nature of pathogens or the expressing tissue [116, 121]. Complements (e.g., *C7* and *Df*) were down-regulated in rainbow trout after viral hemorrhagic septicaemia virus (VHSV) [122]. In zebrafish, the infected individuals showed down-regulation of *C3*, *C8a*, *C8g*, *Crpp* and *Hf* in internal organs after VHSV bath challenge, while up-regulation of many complements at the fins [123]. In addition, the expression levels of factor B and C3 were remarkably lower in the resistant group than in susceptible group of Japanese flounder (*Paralichthys olivaceus*) vaccinated with *Streptococcus iniae* [124]. In this study, the expression of *C3*, *C4–1*, *C5–2*, *C6*, *C7*, D and other complements were down-regulated in all three clones of gibel carp (Additional file 9: Table S6). Moreover, complements and TLR signaling pathways can influence each other in regulating inflammatory responses [125, 126]. Recently, Lebel et al. [127] observed increased immune cell activation and higher production of IFN-α in C3-depleted mice treated by papaya mosaic virus (PapMV)-like nanoparticle, consistent with the activation of IFN system and suppression of complement system in gibel carp after *Ca*HV infection.

A large number of DEUs were mapped to pathway “leukocyte transendothelial migration”, “T cell receptor signaling pathway” and “B cell receptor signaling pathway”. Leukocyte transendothelial migration is a critical step in immune activation. Following immune activation, several chemokines were up-regulated, while chemokine receptors and interleukin receptors were down-regulated their expression after *Ca*HV infection. Chemokines belong to a family of structurally related chemotactic cytokines and regulate migration of monocytes, neutrophils and other effector cells to the sites of tissue infection [128, 129]. The similar dynamical expression changes were observed in other fishes. After bacterial infection with *Edwardsiella ictaluri*, 9 channel catfish (*Ictalurus punctatus*) chemokines were up-regulated [130]. In large yellow croaker (*Pseudosciaena crocea*), *CXCL12* was significantly up-regulated in many tissues after stimulation [131]. In addition, a lot of CD antigens expressed on leukocytes and other cells relevant to the immune system were found to change their expression after *Ca*HV expression. More than 400 CD molecules had been identified in human and classified into about 50 superfamilies, including immunoglobulin superfamily (IgSF), G-protein coupled receptor superfamily, C-type lectin family, cytokine receptor family, TNF superfamily, TNF receptor superfamily, integrin family, tetraspanin family, Toll-like receptor family, cadherin family, and so on [132, 133]. In this case, CD molecules belonging to IgSF (e.g., *CD2*, *CD3*, *CD4*, *CD8*, *CD22*, *CD79b*, *CD80*, *CD86* and *CD276*), cytokine receptor family (e.g., *CCR4*, *CCR5* and *CCR7*), TNF/TNFR superfamily (e.g., *CD40*), integrin family (e.g., *CD11* and *CD18*) and tetraspanin family (e.g., *CD9* and *CD81*) were down-regulated at least in one clone of gibel carp after *Ca*HV infection.

Although the biological functions of CD antigens have been well investigated in mammals, their characterizations in fish remain unclear. Zebrafish (*Danio rerio*) *CD44* and *CD154* were significantly up-regulated after stimulating with KLH [134], while *CD36* was down-regulated during infection of *Mycobacterium marinum* [135]. In rainbow trout, CD antigens in responsive to pathogens are complicated. After VHSV infection or stimulation of *Vibrio* bacterin, *CD28* expression was not significantly different in control and infected fish, while *CD152* was up-regulated in spleenocytes [136], *CD80/86* expression raised in leukocytes [137] and *CD3*, *CD8* and *CD4* increased expression in liver [138] respectively. Additionally, *CD9* and *CD63* were down-regulated in gill or head-kidney by VHSV bath challenge, while they both significantly up-regulated in peritoneal cells when the virus was intraperitoneally injected [139]. In rock bream (*Oplegnathus fasciatus*), the expression dynamical changes of *CD200* depended on pathogens or tissues [140]. Therefore, more studies are needed to confirm the changes of complements, chemokines, and CDs in gibel carp clones.

## Conclusions

This work is the first report to obtain resistant clone against the herpesvirus *Ca*HV and the first transcriptomic comparison between susceptible and resistant clones in gibel carp. Taken together, gibel carp activates IFN system and suppresses complements and CD antigens to defend *Ca*HV invasion. Resistant clone H triggers stronger immune responses with higher expression of significant IFN system genes, IL6 and immunoglobulins, while susceptible clone A^+^ fails to protect from *Ca*HV infection with more expression of apoptosis or death related genes. The further studies of resistance-relevant genes identified in this study could provide useful information for disease control with effective immune protection and for resistance breeding in gibel carp.

## Methods

### Fish

Six month old gibel carps (*Carassius gibelio*) were obtained from the GuanQiao Experimental Station, Institute of Hydrobiology, Chinese academy of sciences, which is located in Wuhan, China. The average weights of clone A^+^, F and H were 85.89 ± 2.13 g, 67.68 ± 2.16 g, 46.96 ± 2.47 g respectively. Apparently healthy individuals were selected to gradually acclimatize in 150-l tanks with aerated water at 24(±1)°C for 2 weeks before infection and fed with commercial feed twice a day. Before infection, 5 individuals of each clone were randomly selected to perform PCR analysis as previously described [74] to confirm *Ca*HV free.

### Transferrin phenotype

Sera was collected from blood of 3 individuals of each clone by centrifugation and treated with rivanol to isolate transferrin, which was applied to 10% polyacrylamide gel electrophoresis (PAGE) following the procedure by Li and Gui [35].

### *Ca*HV infection and sample collection

The *Ca*HV was isolated from the tissues of naturally diseased gibel carp with acute gill hemorrhages. The *Ca*HV was amplified by injection with tissue filtrate from naturally infected fish into healthy gibel carp, identified by PCR assay and electron microscopic observation, and titrated by real-time PCR analysis as previously described [70, 74]. Individuals of each clone were randomly divided into six tanks with 30 fishes per tank. Five tanks of each clone were performed infection with 500 μL *Ca*HV viral suspension (2.915 × 10^8^ virus particles) per fish by intraperitoneal injection. The remaining fishes were injected in equal phosphate-buffered saline solution (PBS) as the control group. Three of the infected tanks were used to record mortality and the rest were used to collect samples. After infection, fish were put back and grown at 24(±1)°C. Water was filtered consecutively and changed daily to keep it visibly clean. Dead fish were taken out timely. Head-kidney tissues were collected from control fishes, infected fishes at 1, 3, 5 and 7dpi, and fishes with sub-clinical and clinical symptoms respectively. The experiment was terminated after 28 days post infection when mortality stabilized. All samples were preserved in RNAlater (QIAGEN) and stored at −20 °C for nucleic acid extraction.

### Histopathology

Head-kidney tissues from 3 individuals of control and diseased fishes with clinical symptoms were fixed in 4% paraformaldehyde over night at 4 °C. After dehydrated and embedded, samples were cut into 4 μm sections and stained with Hematoxylin-Eosin as previously described [70].

### DNA extraction and quantification

Five infected individuals of each clone at 1, 3, 5 and 7 dpi, and diseased fishes with sub-clinical symptoms were selected to evaluate viral load. Total DNA was extracted using DNA extraction kit (Promega, USA) according to the manufacturer’s protocol. Quantification of viral copy numbers were calculated by real-time PCR analysis as previously described [74]. Briefly, a 637-bp helicase gene fragment of *Ca*HV was amplified to serve as the standard for virus quantification. The amplified fragment was purified using a Gel Extraction Kit (OMEGA) and inserted into the pMD18-T plasmid to produce pMD-*Ca*HV (3329 bp). A 10-fold dilution series of pMD-*Ca*HV was used as the standard template of *Ca*HV in the quantitative real-time PCR. The primers and procedure of real-time PCR were designed as described [74].

### RNA extraction and RNA-Seq

In order to eliminate the differences in virus susceptibility among individuals, head-kidney tissues from 3 individuals with sub-clinical symptoms and similar average viral load (10^6^ particles/ng DNA) of each clone were collected and performed the transcriptome analysis. Total RNAs were isolated using SV Total RNA isolation System (Promega, USA) according to the manufacturer’s protocols. The quantity and quality of total RNAs were assessed by Agilent 2100 Bioanalyzer using Agilent RNA 6000 Nano Kit and agarose gel electrophoresis. The subsequent library construction, sequencing and bioinformatics analysis were accomplished by Beijing Genomics Institute (BGI), China. Briefly, total RNAs were digested by Dnase I (NEB) and purified by oligo-dT beads (Invitrogen), then fragmented with Fragment buffer (Ambion). The first strand cDNAs and second strand cDNAs were synthesized successively by First Strand Master Mix and Second Strand Master Mix (Invitrogen). The cDNAs were purified and combined with End Repair Mix. After purified, the end-repaired DNAs were mixed with A-Tailing Mix and combined with Adenylate 3′Ends DNA, Adapter and Ligation Mix. A narrow 300 bp–350 bp size-range DNA fragments were selected and enriched. The final libraries were quantified by real-time qPCR (TaqMan Probe) and the average molecule length determined by using the Agilent 2100 bioanalyzer instrument (Agilent DNA 1000 Reagents). The qualified libraries were amplified on cBot to generate the cluster on the flowcell. Then the amplified flowcells were sequenced pair end on the HiSeq 4000™ System and 150 bp single-end reads were generated.

### Sequence assembly and annotation

After removing the reads with low quality or adaptors, de novo assembly was performed with clean reads to produce the unigenes for downstream bioinformatics analysis. Quality control of clean data was performed through drawing base composition chart and quality distribution chart. Then the unigenes were aligned to NT, NR, COG, KEGG and Swiss-Prot database using Blast, GO database using Blast2GO [141], and InterPro database using InterProScan5 [142]. With the GO and KEGG annotation, unigenes were classified according to official classification and the functional enrichment were performed using phyper, a function of R. False discovery rate (FDR) was used to determine the threshold of *p* value and GO or KEGG terms (FDR ≤ 0.01) were considered significantly enriched.

### Differential expression analysis

To quantify the unigenes expression level, clean reads were mapped to unigenes using Bowtie2 [143] and the expression levels were calculated by RSEM (RNASeq by Expectation Maximization) [144]. Then DEUs were detected with NOIseq based on noisy distribution model [145] and the threshold to judge the significant expression difference was performed by “probability ≥ 0.8 and relative change ≥ 2”. To identify the pathways that DEUs participate in, KEGG enrichment analysis was performed by phyper. Heatmap was generated using MeV and venn diagram was created using jvenn [146].

## Additional files

Additional file 1: Figure S1. Summary of experimental design. (TIFF 423 kb)

Additional file 2: Table S1. Summary statistics of sequencing data. Quality of sequencing reads, transcripts and unigenes are shown respectively. The number 1, 2 and 3 following A^+^, F or H represent three replicate samples of each group. (XLSX 13 kb)

Additional file 3: Table S2. Annotation of all unigenes from gibel carp samples. All of the unigenes were blasted in seven public databases, including NR, NT, GO, COG, KEGG, InterPro and Swiss-Prot database. (XLSX 21424 kb)

Additional file 4: Figure S2. The numbers of differentially expressed unigenes from three comparisons: d-A^+^ vs c-A^+^, d-F vs c-F and d-H vs c-H. The red and green bars indicate up- and down-regulated DEUs respectively. (TIFF 148 kb)

Additional file 5: Table S3. Differentially expressed unigenes (probability ≥0.8 and relative change ≥2) in d-A^+^ vs c-A^+^, d-F vs c-F and d-H vs c-H. List of DEUs includes length, FPKM, log_2_ fold change, *P* value, up- or down-regulation and annotation. (XLSX 3527 kb)

Additional file 6: Figure S3. GO classification of DEUs, which were categorized into 54, 53, 54 terms respectively. GO terms grouped into three main categories: biological process (red bars), cellular component (blue bars) and molecular function (green bars) are showed in the y-axis. The x-axis indicates the numbers of unigenes in each category. (TIFF 4411 kb)

Additional file 7: Table S4. Enriched KEGG pathways in d-A^+^ vs c-A^+^, d-F vs c-F and d-H vs c-H. KEGG pathway description, number of DEUs assigned to the corresponding pathway, *P* value, Q value, pathway ID, KEGG function classification, annotated KO ID of DEUs and unigene ID assigned to the corresponding pathway are shown. (XLSX 398 kb)

Additional file 8: Table S5. Enriched KEGG pathways of DEUs commonly up- and down-regulated in three clones and specifically up- and down-regulated in clone A^+^, F or H. KEGG pathway description, number of DEUs assigned to the corresponding pathway, *P* value, Q value, pathway ID, KEGG function classification, annotated KO ID of DEUs and unigene ID assigned to the corresponding pathway are shown. (XLSX 318 kb)

Additional file 9: Table S6. The common and unique differentially expressed unigenes in d-A^+^ vs c-A^+^, d-F vs c-F and d-H vs c-H. List of DEUs includes FPKM, log_2_ fold change, up- or down-regulation and annotation. (XLSX 1867 kb)

## Abbreviations

COG: Clusters of Orthologous Groups of proteins
DEUs: Differentially expressed unigenes
dpi: days post injection
Gig: Interferon-inducible protein Gig
GO: Gene Ontology
IFI: Interferon-induced protein
IFIT: Interferon-induced protein with tetratricopeptide repeat
IFITM: Interferon induced transmembrane protein
IFN: Interferon
IFNAR: Interferon alpha/beta receptor
IFRD: Interferon-related developmental regulator
Ig: Immunoglobulin
IL: Interleukin
IRF: Interferon regulatory factor
ISG: Interferon stimulated gene
*ISG20L2*: Interferon stimulated exonuclease gene 20-like 2
JAK: Janus kinase
KEGG: Kyoto Encyclopedia of Genes and Genomes
*MAVS*: Mitochondrial antiviral signaling protein
*MDA5*: melanoma differentiation-associated gene
*MITA*: Mediator of IRF3 activation
Mx: Interferon inducible Mx protein
*MyD88*: Myeloid differentiation primary-response protein 88
NCBI: National Center for Biotechnology Information
NR: NCBI non-redundant protein
NT: NCBI non-redundant nucleotide
*PKR*: Double-stranded RNA (dsRNA)-dependent protein kinase
*PKZ*: Protein kinase containing Z-DNA binding domains
*PRKRA*: Protein kinase, interferon-inducible double stranded RNA dependent activator
*PRKRIRA*: Protein-kinase, interferon-inducible double stranded RNA dependent inhibitor, repressor of (P58 repressor) a
*RIG-I*: Retinoic acid-inducible gene I
RNA-Seq: RNA-sequencing
STAT: Signal transducer and activator of transcription
TBK: TANK-binding kinase
TLR: Toll-like receptor
TNFRSF: Tumor necrosis factor receptor superfamily
*TRIM39*: Tripartite motif containing 39
*USP18*: ubiquitin-specific protease 18.

## References

[1] Cherfas NB, Kirpichnikov VS (1981). Gynogenesis in fishes. Genetic bases of fish selection.

[2] Jiang Y-G, Liang S-C, Chen B-D, Yu H-C, Shan S-X, Yang S-E, et al. Biological effect of heterologous sperm on gynogenetic offspring in *Carassius auratus gibelio*. Acta Hydrobiologica Sinica. 1983;1:000.

[3] Kottelat M, Freyhof J (2007). Handbook of European freshwater fish.

[4] Gui J-F, Zhou L (2010). Genetic basis and breeding application of clonal diversity and dual reproduction modes in polyploid *Carassius auratus gibelio*. Sci China Life Sci..

[5] Rylkova K, Kalous L, Slechtova V, Bohlen J (2010). Many branches, one root: first evidence for a monophyly of the morphologically highly diverse goldfish (*Carassius auratus*). Aquaculture.

[6] Li X-Y, Zhang X-J, Li Z, Hong W, Liu W, Zhang J (2014). Evolutionary history of two divergent Dmrt1 genes reveals two rounds of polyploidy origins in gibel carp. Mol Phylogenet Evol..

[7] Liu W, Li S-Z, Li Z, Wang Y, Li X-Y, Zhong J-X (2015). Complete depletion of primordial germ cells in an all-female fish leads to sex-biased gene expression alteration and sterile all-male occurrence. BMC Genomics..

[8] Zhang J, Sun M, Zhou L, Li Z, Liu Z, Li X-Y (2015). Meiosis completion and various sperm responses lead to unisexual and sexual reproduction modes in one clone of polyploid *Carassius gibelio*. Sci Rep..

[9] Zhou L, Wang Y, Gui J-F (2000). Analysis of genetic heterogeneity among five gynogenetic clones of silver crucian carp, *Carassius auratus gibelio* Bloch, based on detection of RAPD molecular markers. Cytogenet Genome Res..

[10] Gui J-F, Zhu Z-Y (2012). Molecular basis and genetic improvement of economically important traits in aquaculture animals. Chinese Sci Bull..

[11] Mei J, Gui J-F (2015). Genetic basis and biotechnological manipulation of sexual dimorphism and sex determination in fish. Sci China Life Sci..

[12] Wang Z-W, Zhu H-P, Wang D, Jiang F-F, Guo W, Zhou L (2011). A novel nucleo-cytoplasmic hybrid clone formed via androgenesis in polyploid gibel carp. BMC Research Notes..

[13] Bureau of Fisheries, Ministry of Agriculture of the People's Republic of China (2016). China fishery statistical yearbook.

[14] Zhou L, Gui J-F. Natural and artificial polyploids in aquaculture. Aquac Fish. 2017.

[15] Zeng X-T, Chen Z-Y, Deng Y-S, Gui J-F, Zhang Q-Y (2016). Complete genome sequence and architecture of crucian carp *Carassius auratus* herpesvirus (*Ca*HV). Arch Virol.

[16] Li L-J, Luo Y-Z, Gao Z-X, Huang J, Zheng X-H, Nie H-H (2015). Molecular characterisation and prevalence of a new genotype of cyprinid herpesvirus 2 in mainland China. Can J Microbiol..

[17] Jung SJ, Miyazaki T (1995). Herpesviral haematopoietic necrosis of goldfish, Carassius auratus (L.). J Fish Dis.

[18] Zhou L, Gui J-F (2002). Karyotypic diversity in polyploid gibel carp, *Carassius auratus gibelio* Bloch. Genetica.

[19] Yi M-S, Li Y-Q, Liu J-D, Zhou L, Yu Q-X, Gui J-F (2003). Molecular cytogenetic detection of paternal chromosome fragments in allogynogenetic gibel carp, *Carassius auratus gibelio Bloch*. Chromosom Res..

[20] Tóth B, Várkonyi E, Hidas A, Meleg EE, Váradi L (2005). Genetic analysis of offspring from intra- and interspecific crosses of *Carassius auratus gibelio* by chromosome and RAPD analysis. J Fish Biol..

[21] Zhu H-P, Ma D-M, Gui J-F (2006). Triploid origin of the gibel carp as revealed by 5S rDNA localization and chromosome painting. Chromosom Res..

[22] Zhu H-P, Gui J-F (2007). Identification of genome organization in the unusual allotetraploid form of *Carassius auratus gibelio*. Aquaculture.

[23] Kalous L, Šlechtová V, Bohlen J, Petrtýl M, Švátora M (2007). First European record of *Carassius langsdorfii* from the Elbe basin. J Fish Biol..

[24] Yang L, Yang S-T, Wei X-H, Gui J-F (2001). Genetic diversity among different clones of the gynogenetic silver crucian carp, *Carassius auratus gibelio*, revealed by transferrin and isozyme markers. Biochem Genet..

[25] Jiang F-F, Wang Z-W, Zhou L, Jiang L, Zhang X-J, Apalikova OV (2013). High male incidence and evolutionary implications of triploid form in northeast Asia *Carassius auratus* complex. Mol Phylogenet Evol..

[26] Zhou L, Wang Y, Gui J-F (2001). Molecular analysis of silver crucian carp (*Carassius auratus gibelio* Bloch) clones by SCAR markers. Aquaculture.

[27] Hänfling B, Bolton P, Harley M, Carvalho GR. A molecular approach to detect hybridisation between crucian carp (*Carassius carassius*) and non-indigenous carp species (*Carassius* spp. and *Cyprinus carpio*). Freshw Biol. 2005;50:403–17.

[28] Vetešník L, Papoušek I, Halačka K, Lusková V, Mendel J (2007). Morphometric and genetic analysis of *Carassius auratus* complex from an artificial wetland in Morava River floodplain. Czech Republic. Fisheries Sci..

[29] Guo W, Gui J-F (2008). Microsatellite marker isolation and cultured strain identification in *Carassius auratus gibelio*. Aquacult Int.

[30] Bai Z-Y, Liu F, Li J-L, Yue G-H (2011). Identification of triploid individuals and clonal lines in *Carassius auratus* complex using microsatellites. Int J Biol Sci..

[31] Jakovlić I, Gui J-F (2011). Recent invasion and low level of divergence between diploid and triploid forms of *Carassius auratus* complex in Croatia. Genetica..

[32] Wouters J, Janson S, Luskova V, Olsen KH (2012). Molecular identification of hybrids of the invasive gibel carp *Carassius auratus* gibelio and crucian carp *Carassius carassius* in Swedish waters. J Fish Biol..

[33] Yang L, Gui J-F (2004). Positive selection on multiple antique allelic lineages of transferrin in the polyploid *Carassius auratus*. Mol Biol Evol..

[34] Yang L, Zhou L, Gui J-F (2004). Molecular basis of transferrin polymorphism in goldfish (*Carassius auratus*). Genetica.

[35] Li F-B, Gui J-F (2008). Clonal diversity and genealogical relationships of gibel carp in four hatcheries. Anim Genet..

[36] Apalikova OV, Eliseikina MG, Kovalev MI, Brykov VA (2008). Collation of data on the ploidy levels and mitochondrial DNA phylogenetic lineages in the silver crucian carp *Carassius auratus gibelio* from far eastern and central Asian populations. Russ J Genet..

[37] Sakai H, Iguchi K, Yamazaki Y, Sideleva V, Goto A (2009). Morphological and mtDNA sequence studies on three crucian carps (*Carassius*: Cyprinidae) including a new stock from the Ob River system. Kazakhstan. J Fish Biol..

[38] Tsipas G, Tsiamis G, Vidalis K, Bourtzis K (2009). Genetic differentiation among Greek lake populations of *Carassius gibelio* and *Cyprinus carpio carpio*. Genetica.

[39] Fan L-C, J-f G, Ding J, Zhu L-F, Liang S-C, Yang Z-A (1997). Cytological mechanism on the integration of heterologous genome or chromosomes in the unique gynogenetic *Carassius auratus gibelio*. Dev Reprod Biol..

[40] Gui J-F. Retrospects and prospects of studies on the mechanism of natural gynogenesis in silver crucian carp (*Carassius auratus gibelio*). Bulletin of National Science Foundation of China 1997; 1:003.

[41] Gui J-F (2015). Scientific frontiers and hot issues in hydrobiology. Chinese Sci Bull..

[42] Wiegertjes GF, Stet RJM, Parmentier HK, Muiswinkel WBV (1996). Immunogenetics of disease resistance in fish: a comparative approach. Dev Comp Immunol..

[43] Leeds TD, Silverstein JT, Weber GM, Vallejo RL, Palti Y, Rexroad CE (2010). Response to selection for bacterial cold water disease resistance in rainbow trout. J Anim Sci..

[44] Wiens GD, Vallejo RL, Leeds TD, Palti Y, Hadidi S, Liu S (2013). Assessment of genetic correlation between bacterial cold water disease resistance and spleen index in a domesticated population of rainbow trout: identification of QTL on chromosome Omy19. Plos One..

[45] Fuji K, Hasegawa O, Honda K, Kumasaka K, Sakamoto T, Okamoto N (2007). Marker-assisted breeding of a lymphocystis disease-resistant Japanese flounder (*Paralichthys olivaceus*). Aquaculture.

[46] Whyte SK (2007). The innate immune response of finfish–a review of current knowledge. Fish Shellfish Immunol..

[47] Zhang Y-B, Gui J-F (2012). Molecular regulation of interferon antiviral response in fish. Dev Comp Immunol..

[48] Krasnov A, Kileng Ø, Skugor S, Jørgensen SM, Afanasyev S, Timmerhaus G (2013). Genomic analysis of the host response to nervous necrosis virus in Atlantic cod (*Gadus morhua*) brain. Mol Immunol..

[49] Shi Y, Zhang Y-B, Zhao Z, Jiang J, Zhang Q-Y, Gui J-F (2008). Molecular characterization and subcellular localization of Carassius auratus interferon regulatory factor-1. Dev Comp Immunol..

[50] Sun F, Zhang Y-B, Liu T-K, Gan L, Yu F-F, Liu Y (2010). Characterization of fish IRF3 as an IFN-inducible protein reveals evolving regulation of IFN response in vertebrates. J Immunol..

[51] Zhang Y-B, Hu C-Y, Zhang J, Huang G-P, Wei L-H, Zhang Q-Y (2003). Molecular cloning and characterization of crucian carp (*Carassius auratus* L.) interferon regulatory factor 7. Fish Shellfish Immunol.

[52] Shi J, Zhang Y-B, Liu T-K, Sun F, Gui J-F (2012). Subcellular localization and functional characterization of a fish IRF9 from crucian carp *Carassius auratus*. Fish Shellfish Immunol..

[53] Zhang Y-B, Gui J-F (2004). Molecular characterization and IFN signal pathway analysis of *Carassius auratus CaSTAT1* identified from the cultured cells in response to virus infection. Dev Comp Immunol..

[54] Sun F, Zhang Y-B, Liu T-K, Shi J, Wang B, Gui J-F (2011). Fish MITA serves as a mediator for distinct fish IFN gene activation dependent on IRF3 or IRF7. J Immunol.

[55] Zhang Y-B, Li Q, Gui J-F (2004). Differential expression of two *Carassius auratus* Mx genes in cultured CAB cells induced by grass carp hemorrhage virus and interferon. Immunogenetics.

[56] Liu T-K, Zhang Y-B, Liu Y, Sun F, Gui J-F (2011). Cooperative roles of fish PKZ and PKR in IFN-mediated antiviral response. J Virol.

[57] Hu C-Y, Zhang Y-B, Huang G-P, Zhang Q-Y, Gui J-F (2004). Molecular cloning and characterisation of a fish PKR-like gene from cultured CAB cells induced by UV-inactivated virus. Fish Shellfish Immunol..

[58] Zhang Y-B, Gui J-F (2004). Identification and expression analysis of two IFN-inducible genes in crucian carp (*Carassius auratus* L.). Gene.

[59] Zhang Y-B, Gui J-F (2004). Identification of two novel interferon-stimulated genes from cultured CAB cells induced by UV-inactivated grass carp hemorrhage virus. Dis Aquat Org..

[60] Zhang Y-B, Wang Y-L, Gui J-F (2007). Identification and characterization of two homologues of interferon-stimulated gene ISG15 in crucian carp. Fish Shellfish Immunol..

[61] Wang B, Zhang Y-B, Liu T-K, Gui J-F (2014). Sequence analysis and subcellular localization of crucian carp *Carassius auratus* viperin. Fish Shellfish Immunol..

[62] Chen C, Zhang Y-B, Gui J-F (2015). Expression characterization, genomic structure and function analysis of fish ubiquitin-specific protease 18 (USP18) genes. Dev Comp Immunol..

[63] Zhang Y-B, Jiang J, Chen Y-D, Zhu R, Shi Y, Zhang Q-Y (2007). The innate immune response to grass carp hemorrhagic virus (GCHV) in cultured *Carassius auratus* blastulae (CAB) cells. Dev Comp Immunol..

[64] Jiang J, Zhang Y-B, Li S, Yu F-F, Sun F, Gui J-F (2009). Expression regulation and functional characterization of a novel interferon inducible gene Gig2 and its promoter. Mol Immunol.

[65] Hao C-Y, Xia Z-Q, Fan R, Tan L-H, Hu L-S, Wu B-D, et al. De novo transcriptome sequencing of black pepper (*Piper nigrum* L.) and an analysis of genes involved in phenylpropanoid metabolism in response to *Phytophthora capsici*. BMC Genomics. 2016;17:822. 10.1186/s12864-016-3155-7 PMC5075214 27769171

[66] Liu Y-J, Guo Y-L, Ma C-Y, Zhang D-F, Wang C, Yang Q (2016). Transcriptome analysis of maize resistance to *Fusarium graminearum*. BMC Genomics.

[67] Matić S, Bagnaresi P, Biselli C, Carneiro GA, Siciliano I, Valé G (2016). Comparative transcriptome profiling of resistant and susceptible rice genotypes in response to the seedborne pathogen *Fusarium fujikuroi*. BMC Genomics..

[68] Polinski MP, Bradshaw JC, Inkpen SM, Richard J, Fritsvold C, Poppe TT (2016). De novo assembly of sockeye salmon kidney transcriptomes reveal a limited early response to piscine reovirus with or without infectious hematopoietic necrosis virus superinfection. BMC Genomics..

[69] Zuluaga AP, Solé M, Lu H-B, Góngora-Castillo E, Vaillancourt B, Coll N, et al. Transcriptome responses to *Ralstonia solanacearum* infection in the roots of the wild potato *Solanum commersonii*. BMC Genomics. 2015;16:1. 10.1186/s12864-015-1460-1 PMC4391584 25880642

[70] Fang J, Deng Y-S, Wang J, Li T, Chen Z-Y, Zhou L (2016). Pathological changes of acute viral hemorrhages in the gills of crucian carp. J Fishery Sci China..

[71] Grabherr MG, Haas BJ, Yassour M, Levin JZ, Thompson DA, Amit I (2011). Full-length transcriptome assembly from RNA-Seq data without a reference genome. Nat Biotechnol..

[72] Pertea G, Huang X-Q, Liang F, Antonescu V, Sultana R, Karamycheva S (2003). TIGR gene indices clustering tools (TGICL): a software system for fast clustering of large EST datasets. Bioinformatics.

[73] Zhai Y-H, Zhou L, Wang Y, Wang Z-W, Li Z, Zhang X-J (2014). Proliferation and resistance difference of a liver-parasitized myxosporean in two different gynogenetic clones of gibel carp. Parasitol Res..

[74] Xu L-J, Lu L-Q, Podok P, Xie J, Lu L-Q (2014). Comparative analysis of differential gene expression in kidney tissues of moribund and surviving crucian carp (*Carassius auratus gibelio*) in response to cyprinid herpesvirus 2 infection. Arch Virol.

[75] Ito T, Kurita J, Ozaki A, Sano M, Fukuda H, Ototake M (2013). Growth of cyprinid herpesvirus 2 (CyHV-2) in cell culture and experimental infection of goldfish *Carassius auratus*. Dis Aquat Org..

[76] Groff JM, Lapatra SE, Munn RJ, Zinkl JG (1998). A viral epizootic in cultured populations of juvenile goldfish due to a putative herpesvirus etiology. J Vet Diagn Investig..

[77] Goodwin AE, Merry GE, Sadler J (2006). Detection of the herpesviral hematopoietic necrosis disease agent (cyprinid herpesvirus 2) in moribund and healthy goldfish: validation of a quantitative PCR diagnostic method. Dis Aquat Org..

[78] Stephens FJ, Raidal SR, Jones B (2004). Haematopoietic necrosis in a goldfish (*Carassius auratus*) associated with an agent morphologically similar to herpesvirus. Aust Vet J..

[79] Jeffery KR, Bateman K, Bayley A, Feist SW, Hulland J, Longshaw C, et al. Isolation of a cyprinid herpesvirus 2 from goldfish, *Carassius auratus* (L.), in the UK. J Fish Dis. 2007;30:649–56. 10.1111/j.1365-2761.2007.00847.x 17958608

[80] Hine PM, Tham KM, Morrison R (2006). Cyprinid herpesvirus 2 in New Zealand goldfish. Theatr Surv.

[81] Doszpoly A, Benko M, Csaba G, Dan A, Lang M, Harrach B (2011). Introduction of the family Alloherpesviridae: the first molecular detection of herpesviruses of cyprinid fish in Hungary. Magy Allatorvosok.

[82] Ito T, Maeno Y (2014). Susceptibility of Japanese Cyprininae fish species to cyprinid herpesvirus 2 (CyHV-2). Vet Microbiol.

[83] Workenhe ST, Rise ML, Kibenge MJT, Kibenge FSB (2010). The fight between the teleost fish immune response and aquatic viruses. Mol Immunol.

[84] Zhang Q-Y, Gui J-F (2015). Virus genomes and virus-host interactions in aquaculture animals. Sci China Life Sci..

[85] Ellis A (2001). Innate host defense mechanisms of fish against viruses and bacteria. Dev Comp Immunol..

[86] Sadler AJ, Williams BR (2008). Interferon-inducible antiviral effectors. Nat Rev Immunol..

[87] Verrier ER, Langevin C, Benmansour A, Boudinot P (2011). Early antiviral response and virus-induced genes in fish. Dev Comp Immunol..

[88] Zou J, Secombes CJ (2011). Teleost fish interferons and their role in immunity. Dev Comp Immunol..

[89] Zhu L-Y, Nie L, Zhu G, Xiang L-X, Shao J-Z (2013). Advances in research of fish immune-relevant genes: a comparative overview of innate and adaptive immunity in teleosts. Dev Comp Immunol..

[90] Cofre C, Gonzalez R, Moya J, Vidal R (2014). Phenotype gene expression differences between resistant and susceptible salmon families to IPNV. Fish Physiol Biochem.

[91] Reyes-López FE, Romeo JS, Vallejos-Vidal E, Reyes-Cerpa S, Sandino AM, Tort L (2015). Differential immune gene expression profiles in susceptible and resistant full-sibling families of Atlantic salmon (*Salmo salar*) challenged with infectious pancreatic necrosis virus (IPNV). Dev Comp Immunol..

[92] Robledo D, Taggart JB, Ireland JH, McAndrew BJ, Starkey WG, Haley CS (2016). Gene expression comparison of resistant and susceptible Atlantic salmon fry challenged with infectious pancreatic necrosis virus reveals a marked contrast in immune response. BMC Genomics..

[93] Rakus KL, Wiegertjes GF, Adamek M, Siwicki AK, Lepa A, Irnazarow I. Resistance of common carp (Cyprinus carpio L.) to cyprinid herpesvirus-3 is influenced by major histocompatibility (MH) class II B gene polymorphism. Fish Shellfish Immunol. 2009;26:737-43. 10.1016/j.fsi.2009.03.001 19328856

[94] Kongchum P, Sandel E, Lutzky S, Hallerman EM, Hulata G, David L (2011). Association between IL-10a single nucleotide polymorphisms and resistance to cyprinid herpesvirus-3 infection in common carp (*Cyprinus carpio*). Aquaculture..

[95] Savan R, Sakai M (2006). Genomics of fish cytokines. Comp Biochem Phys D..

[96] Zou J, Secombes CJ (2016). The function of fish cytokines. Biology.

[97] Hirano T (1998). Interleukin 6 and its receptor: ten years later. Int Rev Immunol..

[98] Bird S, Zou J, Savan R, Kono T, Sakai M, Woo J (2005). Characterisation and expression analysis of an interleukin 6 homologue in the Japanese pufferfish, *Fugu Rubripes*. Dev Comp Immunol..

[99] Iliev DB, Castellana B, MacKenzie S, Planas JV, Goetz FW (2007). Cloning and expression analysis of an IL-6 homolog in rainbow trout (*Oncorhynchus mykiss*). Mol Immunol.

[100] Nam BH, Byon JY, Kim YO, Park EM, Cho YC, Cheong JH (2007). Molecular cloning and characterisation of the flounder (*Paralichthys olivaceus*) interleukin-6 gene. Fish Shellfish Immunol..

[101] Castellana B, Iliev DB, Sepulcre MP, MacKenzie S, Goetz FW, Mulero V (2008). Molecular characterization of interleukin-6 in the gilthead seabream (*Sparus aurata*). Mol Immunol..

[102] Neves JV, Caldas C, Wilson JM, Rodrigues PNS (2011). Molecular mechanisms of hepcidin regulation in sea bass (*Dicentrarchus labrax*). Fish Shellfish Immunol..

[103] Wang T-H, Holland JW, Bols N, Secombes CJ (2005). Cloning and expression of the first nonmammalian interleukin-11 gene in rainbow trout *Oncorhynchus mykiss*. FEBS J.

[104] Santos MD, Yasuike M, Kondo H, Hirono I, Aoki T (2008). Teleostean IL11b exhibits complementing function to IL11a and expansive involvement in antibacterial and antiviral responses. Mol Immunol.

[105] Costa MM, Maehr T, Diaz-Rosales P, Secombes CJ, Wang T-H (2011). Bioactivity studies of rainbow trout (*Oncorhynchus mykiss*) interleukin-6: effects on macrophage growth and antimicrobial peptide gene expression. Mol Immunol.

[106] Kopf M, Baumann H, Freer G, Freudenberg M, Lamers M, Kishimoto T (1994). Impaired immune and acute-phase responses in interleukin-6-deficient mice. Nature..

[107] Kaneda M, Odaka T, Suetake H, Tahara D, Miyadai T (2012). Teleost IL-6 promotes antibody production through STAT3 signaling via IL-6R and gp130. Dev Comp Immunol..

[108] Chen HH, Lin HT, Foung YF, Lin JHY (2012). The bioactivity of teleost IL-6: IL-6 protein in orange-spotted grouper (*Epinephelus coioides*) induces Th2 cell differentiation pathway and antibody production. Dev Comp Immunol..

[109] Fiorentino DF, Zlotnik A, Mosmann TR, Howard M, O'garra A (1991). IL-10 inhibits cytokine production by activated macrophages. J Immunol.

[110] Di Carlo E, Coletti A, Modesti A, Giovarelli M, Forni G, Musiani P (1998). Local release of interleukin-10 by transfected mouse adeno-carcinoma cells exhibits pro-and anti-inflammatory activity and results in a delayed tumor rejection. Eur Cytokine Netw..

[111] Karan S, Dash P, Kaushik H, Sahoo PK, Garg LC, Dixit A. Structural and functional characterization of recombinant interleukin-10 from Indian major carp Labeo rohita. J Immunol Res. 2016;2016. 10.1155/2016/3962596 PMC5023823 27689097

[112] Tamura T, Ishihara M, Lamphier MS, Tanaka N, Oishi I, Aizawa S (1995). An IRF-1-dependent pathway of DNA damage-induced apoptosis in mitogen-activated T lymphocytes. Nature..

[113] Lambris JD, Ricklin D, Geisbrecht BV (2008). Complement evasion by human pathogens. Nat Rev Microbiol..

[114] Ojha H, Panwar HS, Gorham RD, Morikis D, Sahu A (2014). Viral regulators of complement activation: structure, function and evolution. Mol Immunol.

[115] Rettig TA, Harbin JN, Harrington A, Dohmen L, Fleming SD (2015). Evasion and interactions of the humoral innate immune response in pathogen invasion, autoimmune disease, and cancer. Clin Immunol.

[116] Nakao M, Tsujikura M, Ichiki S, Vo TK, Somamoto T (2011). The complement system in teleost fish: progress of post-homolog-hunting researches. Dev Comp Immunol..

[117] Mackay IR, Rosen FS, Walport MJ (2001). Complement, first of two parts. N Engl J med.

[118] Le Friec G, Kemper C (2009). Complement: coming full circle. Arch Immunol Ther Ex..

[119] Mei J, Gui J-F (2008). Bioinformatic identification of genes encoding C1q-domain-containing proteins in zebrafish. J Genet Genomics..

[120] Mei J, Zhang Q-Y, Li Z, Lin S, Gui J-F (2008). C1q-like inhibits p53-mediated apoptosis and controls normal hematopoiesis during zebrafish embryogenesis. Dev Biol..

[121] Boshra H, Li J, Sunyer JO (2006). Recent advances on the complement system of teleost fish. Fish Shellfish Immunol..

[122] Jørgensen HBH, Sørensen P, Cooper G, Lorenzen E, Lorenzen N, Hansen MH (2011). General and family-specific gene expression responses to viral hemorrhagic septicaemia virus infection in rainbow trout (*Oncorhynchus mykiss*). Mol Immunol..

[123] Encinas P, Rodriguez-Milla MA, Novoa B, Estepa A, Figueras A, Coll J (2010). Zebrafish fin immune responses during high mortality infections with viral haemorrhagic septicemia rhabdovirus. A proteomic and transcriptomic approach. BMC Genomics..

[124] Yasuike M, Kondo H, Hirono I, Aoki T (2007). Difference in Japanese flounder, *Paralichthys Olivaceus* gene expression profile following hirame rhabdovirus (HIRRV) G and N protein DNA vaccination. Fish Shellfish Immunol..

[125] Hajishengallis G, Lambris JD (2010). Crosstalk pathways between toll-like receptors and the complement system. Trends Immunol.

[126] Hajishengallis G, Lambris JD (2016). More than complementing tolls: complement–toll-like receptor synergy and crosstalk in innate immunity and inflammation. Immunol Rev..

[127] Lebel M-È, Langlois M-P, Daudelin J-F, Tarrab E, Savard P, Leclerc D (2017). Complement component 3 regulates IFN-α production by plasmacytoid dendritic cells following TLR7 activation by a plant virus–like nanoparticle. J Immunol..

[128] Ransohoff RM (2009). Chemokines and chemokine receptors: standing at the crossroads of immunobiology and neurobiology. Immunity..

[129] Salazar-Mather TP, Hokeness KL, Lane TE (2006). Cytokine and chemokine networks: pathways to antiviral defense. Chemokines and viral infection.

[130] Peatman E, Bao B-L, Peng X, Baoprasertkul P, Brady Y, Liu Z-J (2006). Catfish CC chemokines: genomic clustering, duplications, and expression after bacterial infection with *Edwardsiella Ictaluri*. Mol Gen Genomics..

[131] Wan X, Chen X-H (2009). Molecular cloning and expression analysis of a CXC chemokine gene from large yellow croaker *Pseudosciaena crocea*. Vet Immunol Immunop.

[132] Engel P, Boumsell L, Balderas R, Bensussan A, Gattei V, Horejsi V (2015). CD nomenclature 2015: Human Leukocyte Differentiation Antigen Workshops as a Driving Force in Immunology. J Immunol..

[133] Clark G, Stockinger H, Balderas R, van Zelm MC, Zola H, Hart D (2016). Nomenclature of CD molecules from the Tenth Human Leucocyte Differentiation Antigen Workshop. Clinical & Translational Immunology..

[134] Gong Y-F, Xiang L-X, Shao J-Z (2009). CD154-CD40 interactions are essential for thymus-dependent antibody production in zebrafish: insights into the origin of costimulatory pathway in helper T cell-regulated adaptive immunity in early vertebrates. J Immunol.

[135] Fink IR, Benard EL, Hermsen T, Meijer AH, Forlenza M, Wiegertjes GF (2015). Molecular and functional characterization of the scavenger receptor CD36 in zebrafish and common carp. Mol Immunol.

[136] Bernard D, Riteau B, Hansen JD, Phillips RB, Michel F, Boudinot P (2006). Costimulatory receptors in a teleost fish: typical CD28, elusive CTLA4. J Immunol.

[137] Zhang Y-A, J-i H, Li J, SE LP, Luo Y-P, Sunyer JO (2009). Conservation of structural and functional features in a primordial CD80/86 molecule from rainbow trout (*Oncorhynchus mykiss*), a primitive teleost fish. J Immunol.

[138] Castro R, Abós B, Pignatelli J, von Gersdorff JL, Granja AG, Buchmann K (2014). Early immune responses in rainbow trout liver upon viral hemorrhagic septicemia virus (VHSV) infection. Plos One..

[139] Castro R, Abós B, González L, Aquilino C, Pignatelli J, Tafalla C (2015). Molecular characterization of CD9 and CD63, two tetraspanin family members expressed in trout B lymphocytes. Dev Comp Immunol..

[140] Hwang SD, Kim JW, Kim MC, Kim DH, Park CI (2013). First molecular cloning and gene expression analysis of a teleost CD200 (OX-2) glycoprotein from rock bream. Oplegnathus fasciatus. Fish Shellfish Immunol..

[141] Conesa A, Gotz S, Garcia-Gomez JM, Terol J, Talon M, Robles M (2005). Blast2GO: a universal tool for annotation, visualization and analysis in functional genomics research. Bioinformatics.

[142] Quevillon E, Silventoinen V, Pillai S, Harte N, Mulder N, Apweiler R, et al. InterProScan: protein domains identifier. Nucleic Acids Res. 2005;33:W116–W20. 10.1093/nar/gki442 PMC1160203 15980438

[143] Langmead B, Salzberg SL (2012). Fast gapped-read alignment with bowtie 2. Nat Methods..

[144] Li B, Dewey CN. RSEM: accurate transcript quantification from RNA-Seq data with or without a reference genome. BMC Bioinformatics. 2011;12. 10.1186/1471-2105-12-323 PMC3163565 21816040

[145] Tarazona S, Garcia-Alcalde F, Dopazo J, Ferrer A, Conesa A (2011). Differential expression in RNA-seq: a matter of depth. Genome Res..

[146] Bardou P, Mariette J, Escudie F, Djemiel C, Klopp C (2014). Jvenn: an interactive Venn diagram viewer. BMC Bioinformatics..

