# Supplementary figures and images for: Distinct herpesvirus resistances and immune responses of three gynogenetic clones of gibel carp revealed by comprehensive transcriptomes
Source: BMC Genomics. 2017 Jul 24;18:561. doi: 10.1186/s12864-017-3945-6 (PMC5525251; doi:10.1186/s12864-017-3945-6)

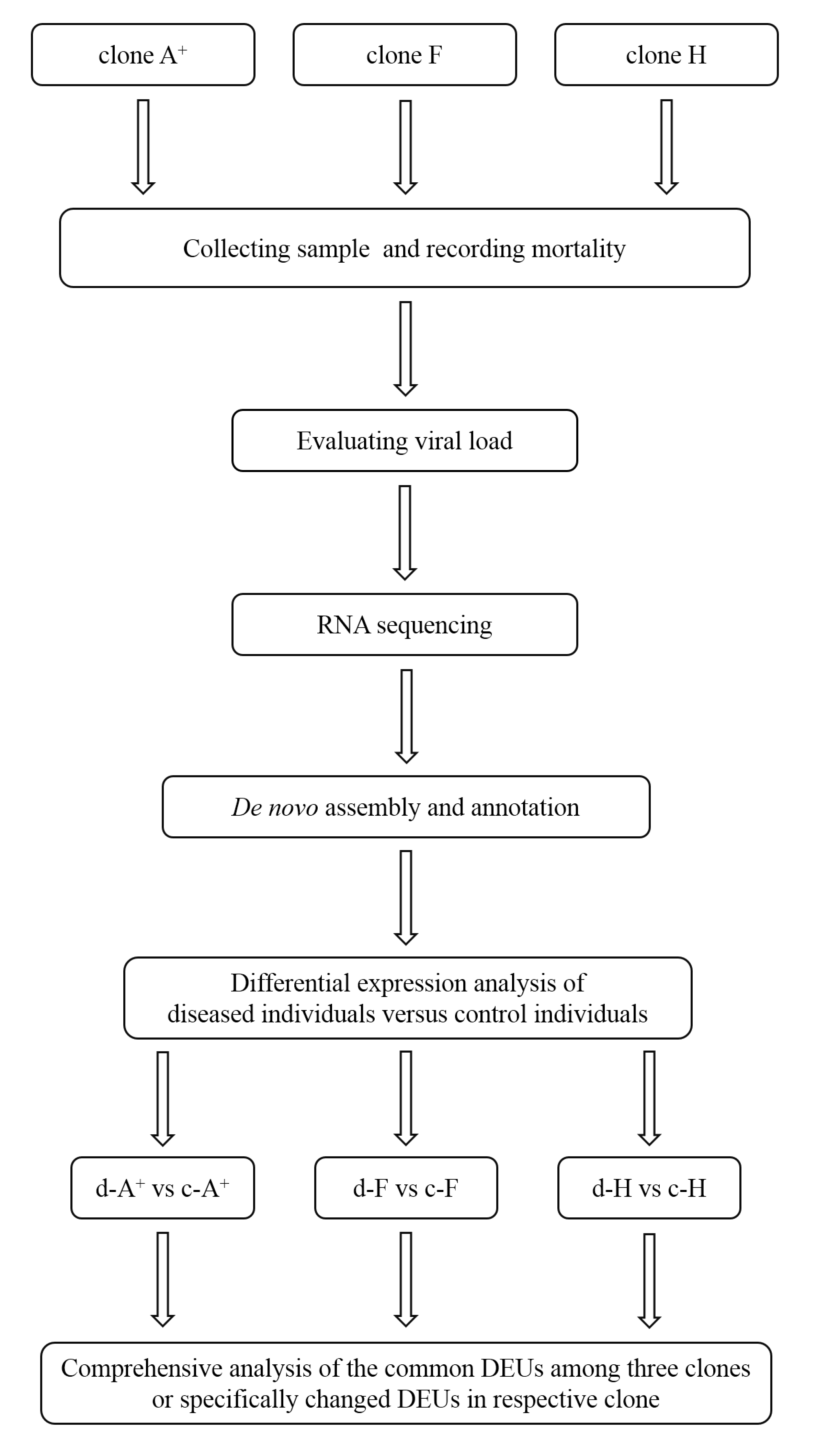

Supplement: Supplementary file 1 — Summary of experimental design. (TIFF 423 kb) [file 12864_2017_3945_MOESM1_ESM.tif]

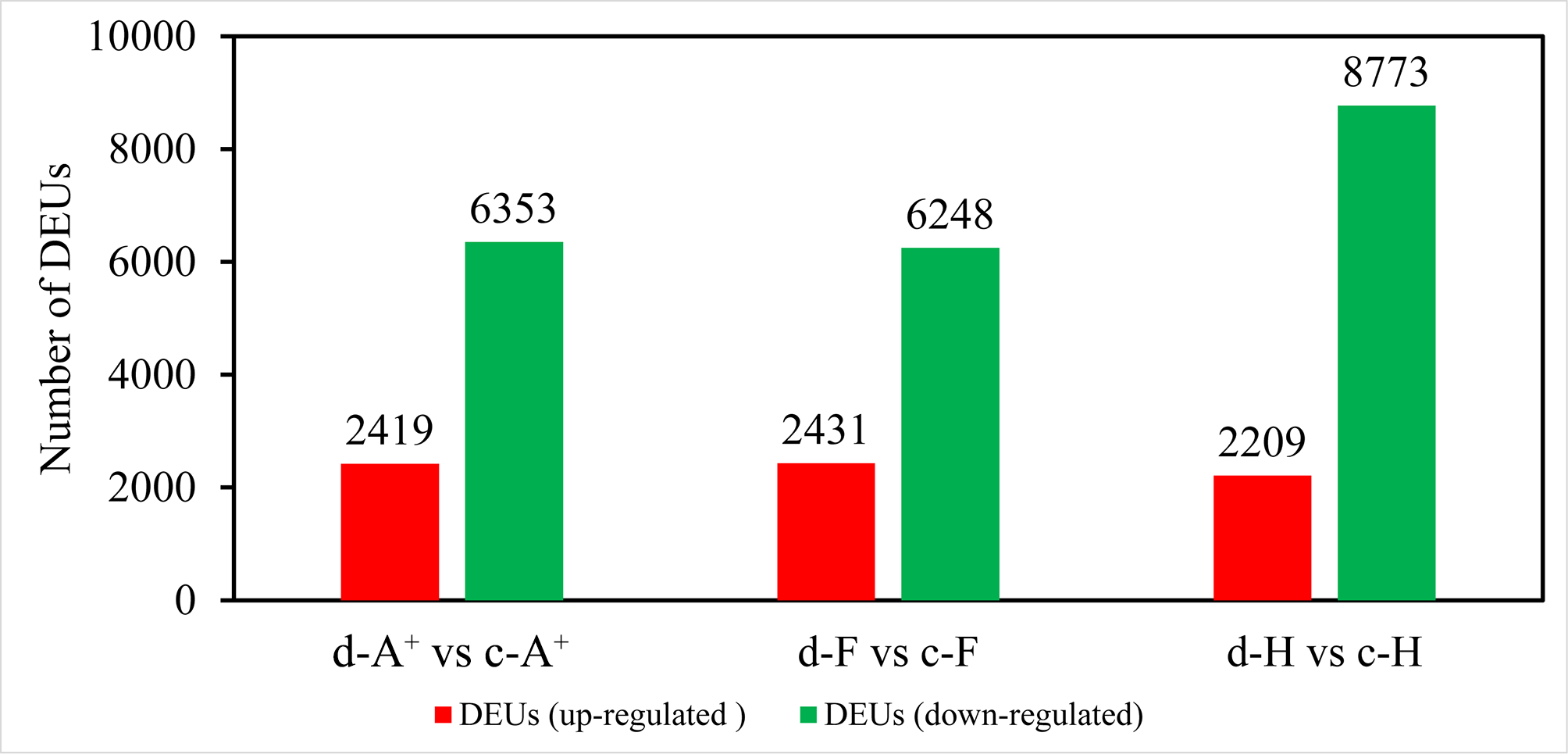

Supplement: Supplementary file 4 — The numbers of differentially expressed unigenes from three comparisons: d-A+ vs c-A+, d-F vs c-F and d-H vs c-H. The red and green bars indicate up- and down-regulated DEUs respectively. (TIFF 148 kb) [file 12864_2017_3945_MOESM4_ESM.tif]

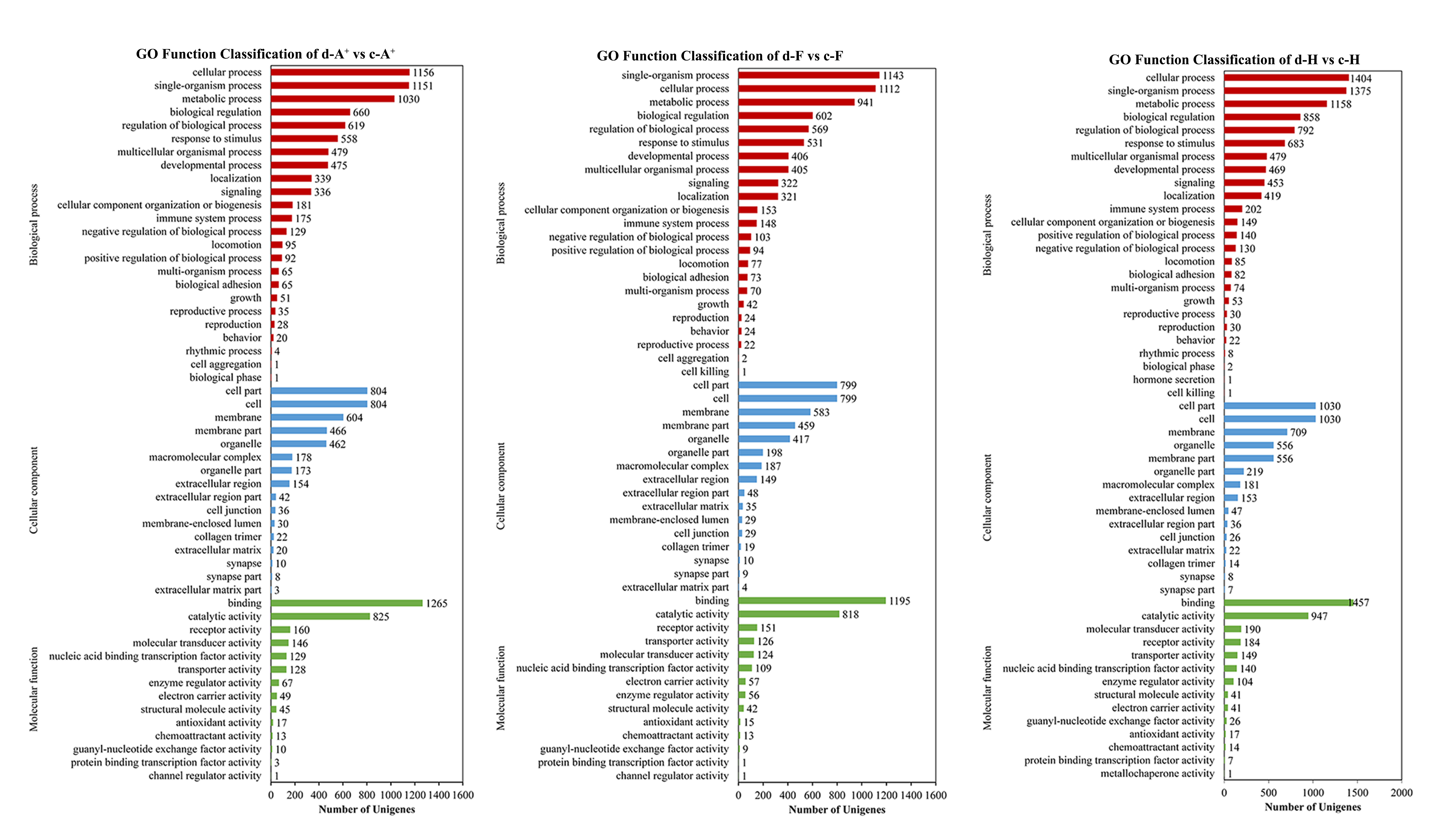

Supplement: Supplementary file 6 — GO classification of DEUs, which were categorized into 54, 53, 54 terms respectively. GO terms grouped into three main categories: biological process (red bars), cellular component (blue bars) and molecular function (green bars) are showed in the y-axis. The x-axis indicates the numbers of unigenes in each category. (TIFF 4411 kb) [file 12864_2017_3945_MOESM6_ESM.tif]
